# Germanium Monosulfide as a Natural Platform for Highly Anisotropic THz Polaritons

**DOI:** 10.1021/acsnano.2c05376

**Published:** 2022-11-29

**Authors:** Tobias Nörenberg, Gonzalo Álvarez-Pérez, Maximilian Obst, Lukas Wehmeier, Franz Hempel, J. Michael Klopf, Alexey Y. Nikitin, Susanne C. Kehr, Lukas M. Eng, Pablo Alonso-González, Thales V. A. G. de Oliveira

**Affiliations:** † Institut für Angewandte Physik, Technische Universität Dresden, Dresden 01187, Germany; ‡ Würzburg-Dresden Cluster of Excellence - EXC 2147 (ct.qmat), Dresden 01062, Germany; § Institute of Radiation Physics, Helmholtz-Zentrum Dresden-Rossendorf, Dresden 01328, Germany; ∥ Department of Physics, University of Oviedo, Oviedo 33006, Spain; # Center of Research on Nanomaterials and Nanotechnology CINN (CSIC−Universidad de Oviedo), El Entrego 33940, Spain; □ Collaborative Research Center 1415, Technische Universität Dresden, Dresden 01069, Germany; ■ Donostia International Physics Center (DIPC), Donostia-San Sebastián 20018, Spain; △ IKERBASQUE, Basque Foundation for Science, Bilbao 48013, Spain

**Keywords:** van der Waals materials, optical anisotropy, terahertz, phonon polaritons, polariton interferometry, near-field optics

## Abstract

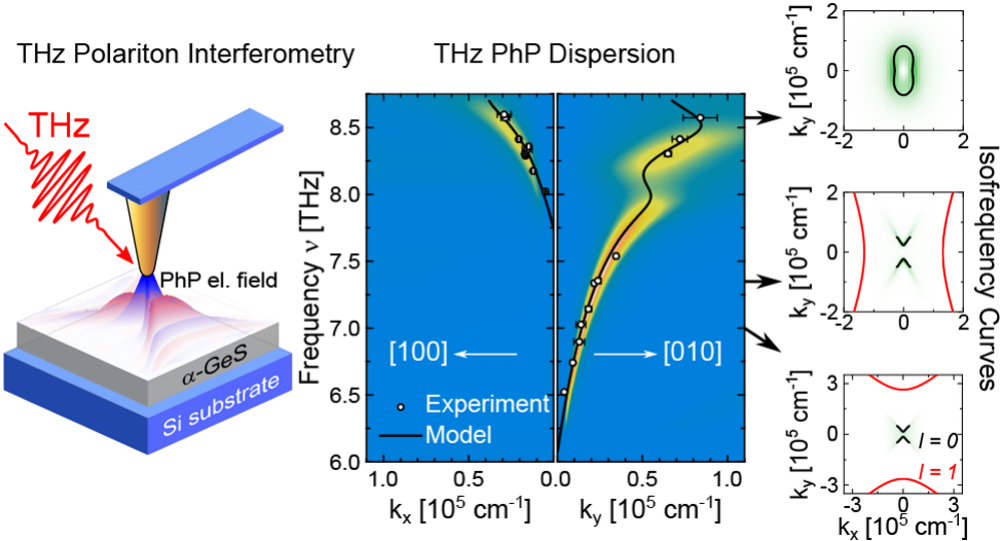

Terahertz (THz) electromagnetic radiation is key to access collective excitations such as magnons (spins), plasmons (electrons), or phonons (atomic vibrations), thus bridging topics between optics and solid-state physics. Confinement of THz light to the nanometer length scale is desirable for local probing of such excitations in low-dimensional systems, thereby circumventing the large footprint and inherently low spectral power density of far-field THz radiation. For that purpose, phonon polaritons (PhPs) in anisotropic van der Waals (vdW) materials have recently emerged as a promising platform for THz nanooptics. Hence, there is a demand for the exploration of materials that feature not only THz PhPs at different spectral regimes but also host anisotropic (directional) electrical, thermoelectric, and vibronic properties. To that end, we introduce here the semiconducting vdW-material alpha-germanium(II) sulfide (GeS) as an intriguing candidate. By employing THz nanospectroscopy supported by theoretical analysis, we provide a thorough characterization of the different in-plane hyperbolic and elliptical PhP modes in GeS. We find not only PhPs with long lifetimes (τ > 2 ps) and excellent THz light confinement (λ_0_/λ > 45) but also an intrinsic, phonon-induced anomalous dispersion as well as signatures of naturally occurring, substrate-mediated PhP canalization within a single GeS slab.

## Introduction

Polaritons refer to quasiparticles formed by light strongly coupled to collective excitations in matter.^1^ The hybrid light–matter nature of polaritons offers a promising platform for the manipulation of the flow of light at the nanoscale.^2^ Notably, phonon polaritons (PhPs) in layered van der Waals (vdW) materials such as hBN, α-MoO_3_, or α-V_2_O_5_ have recently attracted great interest^3−5^ since, apart from featuring field confinement to the nanoscale, they naturally exhibit anisotropic (and particularly directional) propagation, ultralong lifetimes (of several ps), and low group velocities.^6^ Polaritons hold great promise in a manifold of potential applications, such as nanolasers,^7,8^ polarization-sensitive detectors,^9^ molecular sensors,^10^ hyper-lensing,^11,12^ or waveguiding,^13^ and are, thus, key to nanophotonics.^14−16^ However, a significant obstacle to such applications is presented by the PhPs exclusively residing in the polar material’s reststrahlen bands (RB): These spectral regions between the transverse optical (TO) and longitudinal optical (LO) phonon modes are typically located in the mid-infrared (MIR) to THz part of the electromagnetic spectrum, where the negative sign of the permittivity enables the excitation of confined polariton modes.^17,18^ Thus, routes for spectral tunability (e*.*g., ion intercalation,^5^ nanostructuring,^19^ isotopic enrichment,^20^ carrier photoinjection,^21^ or modification of the dielectric environment^22−24^) as well as materials with RBs covering complementary spectral bands are of great need. Especially in the scientifically and technologically emerging THz regime, the direct observation of confined PhP modes remains widely elusive with only few recent works.^25−28^ Note here that ordinary, nonconfined phonon-polariton waves have long been observed in the THz regime,^29^ in spectral regions where the host material’s permittivity is positive.

A promising material class to observe PhPs is presented by highly anisotropic vdW materials, as they can host hyperbolic polariton dispersion resulting in ray-like propagation, enhanced confinement, and recently reported diffraction-less propagation in twisted-bilayer-engineered devices.^30−33^ Furthermore, in contrast to in-plane hyperbolic PhPs in metamaterials, PhPs in natural vdW crystals exhibit significantly lower losses that are not limited by fabrication imperfections.^6^ Yet, the palette of vdW materials supporting nanoscale-confined PhPs in the THz spectral range is very scarce. Here, we add a new member to this palette by introducing the family of group-IV monochalcogenide semiconductor compounds MX (M = Ge, Sn; X = S, Se) as a rich platform for THz nanophotonics. Their layered orthorhombic crystal structure, similar to that of black phosphorus,^34^ gives rise to their strongly anisotropic optical, vibrational, and electrical properties.^35^ In particular, germanium sulfide stands out due to interesting physical properties, such as a direct bandgap of 1.6 eV, thus potentially enabling polaritonic control through electric gating,^34,36^ its characteristic photoluminescence,^37^ an outstanding Seebeck coefficient,^38^ ferroelectricity in twisted nanowires^39,40^ and in the monolayer limit,^41^ resistance to oxidation,^42^ and exciton polaritons at visible wavelengths.^43^

In this study, we focus on the recently predicted^44^ THz PhPs in the semiconductor compound alpha-germanium(II) sulfide (α-GeS, GeS) that exhibit an intriguing polariton dispersion in the frequency range ν = 6.0–9.5 THz. We provide a comprehensive characterization of the rich THz PhP modes including their dispersion, quality factors, lifetimes, and electromagnetic field confinement at the nanometer length scale. To that end, we carry out polariton interferometry experiments by employing a free-electron laser (FEL) as a narrowband THz light source.^25^ Our results, supported by full-wave numerical simulations, as well as transfer matrix and analytical dispersion calculations, unveil THz PhPs with high quality factors (*Q* = 10), long lifetimes (τ > 2 ps), and deep subwavelength confinement (up to λ_0_/45, with λ_0_ the incident free-space light wavelength). Moreover, we predict spectral areas of anomalous PhP dispersion and a related canalized PhP propagation at distinct frequencies, with both effects being strongly substrate dependent.

**Figure 1:**
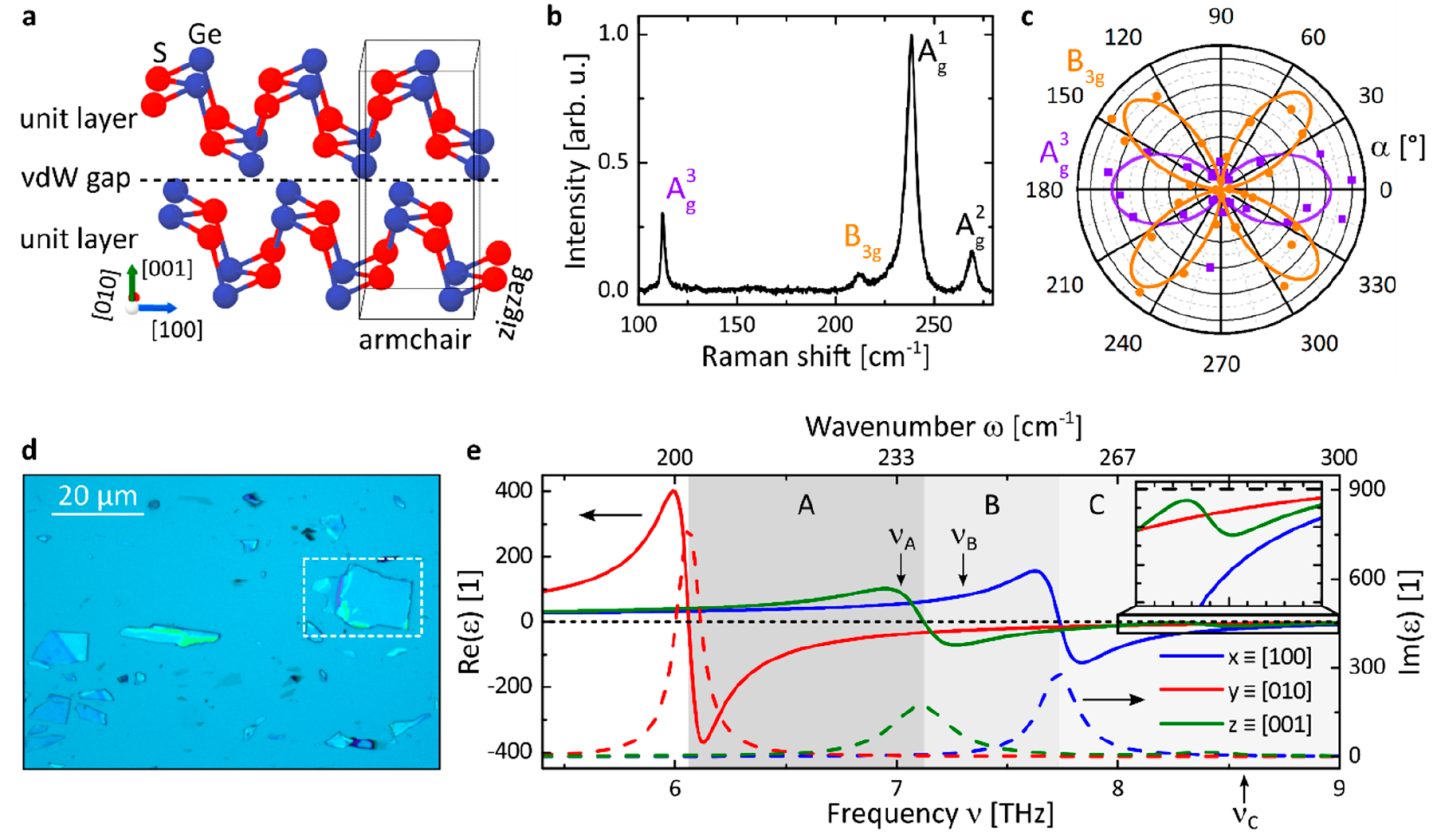
Material properties of germanium sulfide (α-GeS). (a) Crystal structure of α-GeS. The crystal is composed of layers of covalently bonded Ge (blue) and S (red) atoms with the vdW stacking direction along the [001] crystal direction. Similar to black phosphorus, the layers show an armchair and zigzag geometry along the [100] and [010] crystal directions, respectively. The box marks the unit cell. (b) Representative Raman spectrum for an incident linear polarization aligned with the [100] crystal axis. The characteristic polarization dependence of the A_g_^3^ mode at 112 cm^–1^ (purple) and B_3g_ mode at 213 cm^–1^ (orange) can be used for unambiguous identification of the crystal axis orientation. (c) Polar plot of the normalized Raman scattering intensities of the A_g_^3^ and B_3g_ mode in (b) with α = 0° corresponding to the [100] crystal direction. The lobes of the 2-fold rotational symmetric A_g_^3^ mode extend along the [100] crystal direction.^46^ The 4-fold symmetric B_3g_ mode is rotated by 45° relative to the A_g_^3^ mode. (d) Optical microscopy image of exfoliated GeS crystals on silicon. The dashed box marks the flake investigated in this work. (e) Real (solid lines) and imaginary (dashed lines) components of the complex permittivity ε. The permittivity in the THz regime is governed by four optical phonons and exhibits two in-plane reststrahlen bands RB_*y*_ and RB_*x*_. The inset highlights the real part of ε from 8 to 9 THz. The shaded areas A, B, and C identify three spectral regions with a different constitution of Re(ε_*i*_) (*i* = *x, y, z*).

The layered orthorhombic crystal structure of GeS (space group *Pcmn*) is depicted in Figure 1a. In analogy to that of black phosphorus,^34^ it consists of covalently bound layers stacked in the [001] direction with an armchair structure in the [100] direction and zigzag structure in the [010] direction.^35^ The difference in lattice constants (*a* = 4.29 Å, *b* = 3.64 Å, *c* = 10.42 Å) is remarkable, in particular the large ratio *a*/*b* = 1.18 gives rise to a high structural in-plane anisotropy within the layers that is 2.5 times higher than in the likewise biaxial (i.e., ε_*x*_(ω) ≠ ε_*y*_(ω) ≠ ε_*z*_(ω)) α-MoO_3_ crystal (*a*/*c* = 1.072)^45^ in which THz polaritons have been recently demonstrated.^25^ Our micro-Raman spectrum (Figure 1b) unveils four Raman peaks at Raman shifts of 112, 213, 240, and 270 cm^–1^ that can be readily attributed to the A_g_^3^, B_3g_, A_g_^1^, and A_g_^2^ phonon modes, respectively.^37^ Particularly, their polarization dependence allows for deducing the GeS crystal structure orientation of individual flakes. To that end, we find the maximum Raman intensity of the A_g_^3^ mode in Figure 1c (purple; that is parallel to the [100] crystal axis^46^) to be aligned along the right edge for the specific GeS flake marked in the optical microscopy image in Figure 1d.

In addition to the Raman-active phonons, the polar GeS exhibits several well-characterized, directional optical phonons located in the THz spectral regime^35^ that govern its dielectric permittivity ε (Figure 1e). We define the coordinate system to align with the GeS crystallographic axes as *x* ≡ [100], *y* ≡ [010], and *z* ≡ [001]. At frequencies from 6 to 10 THz, the permittivity is negative (*Re*(ε_*i*_) < 0, *i* = *x, y, z*) along different crystal axes within four RBs, with two of them lying in the *x*, *y*-plane: RB_*y*_ (ν_TO*,y*_ = 6.06 THz and ν_LO,*y*_ = 9.47 THz) and RB_*x*_ (ν_TO,*x*_ = 7.74 THz and ν_LO*,x*_ = 9.65 THz). Along the *z*-direction, GeS exhibits two out-of-plane TO phonons (ν_TO,*z*,1_ = 7.1 THz and ν_TO,*z*,2_ = 8.4 THz) that spectrally overlap with the in-plane RBs, giving rise to an exotic, highly anisotropic optical response. Consequently, the considered permittivity regime may be classified into three distinct spectral areas, A–C (as shaded in Figure 1e), that hold a differently constituted *Re*(ε):

- Area A (ν = 6.06–7.1 THz), with *Re*(ε_*y*_) < 0 and *Re*(ε_*x*_, ε_*z*_) > 0;
- Area B (ν = 7.1–7.74 THz), with *Re*(ε_*y*_, ε_*z*_) < 0 and *Re*(ε_*x*_) > 0;
- Area C (ν = 7.74–9.47 THz), with *Re*(ε_*x*_, ε_*y*_, ε_*z*_) < 0.

Within each of the three areas, we select a representative frequency ν_*i*_ (*i* = A, B, C), for which detailed experimental and theoretical data will be presented in this work.

## Results and Discussion

### Polariton Interferometry Experiment

**Figure 2:**
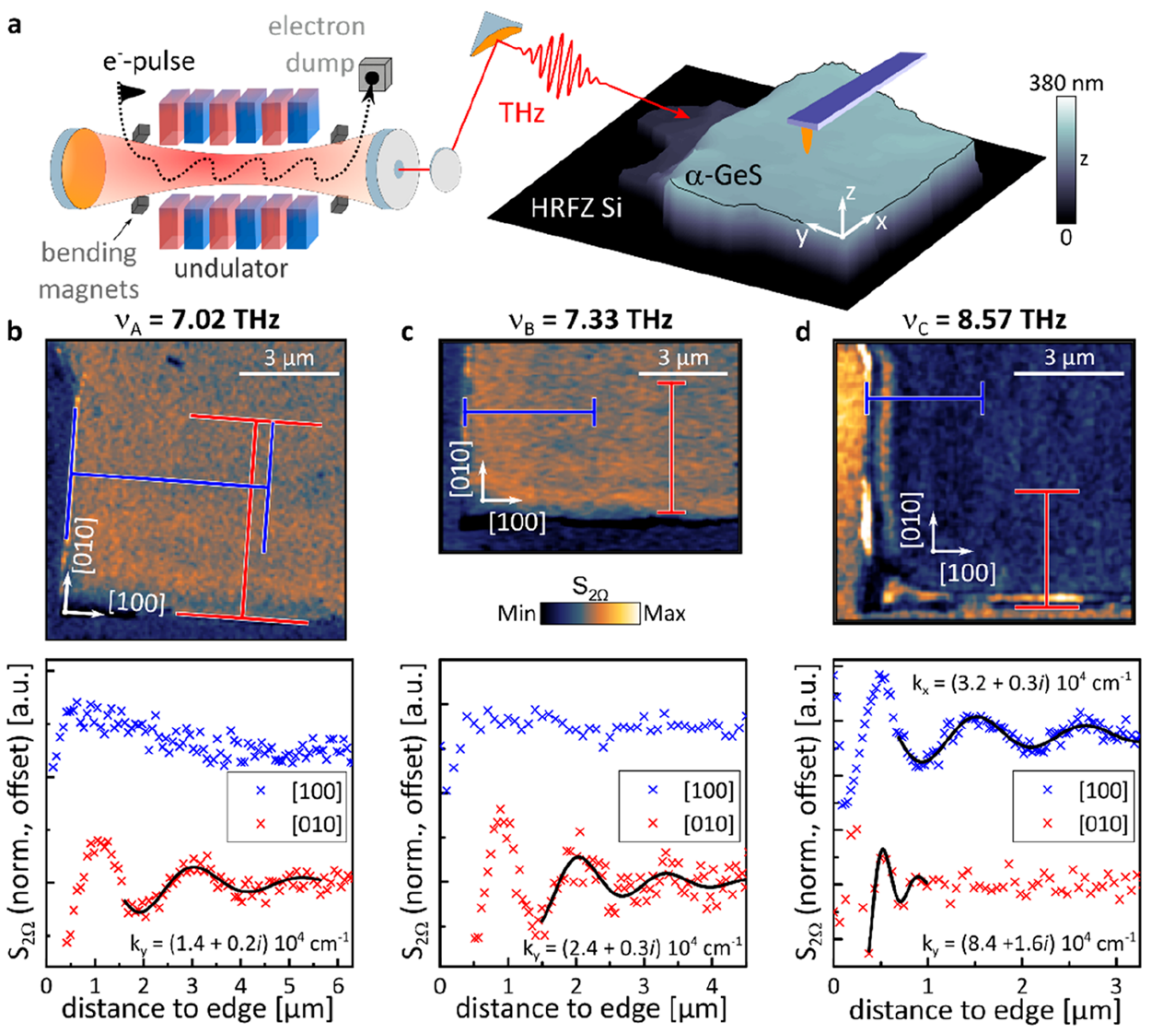
Polariton interferometry of THz PhPs in GeS. (a) Schematic of the experimental setup. The AFM tip excited by the FEL’s THz radiation launches PhPs that propagate across a 224 nm thick GeS slab. (b, c, d) Optical near-field intensity *S*_2Ω_ images of the bottom right corner of the slab (top) and corresponding *S*_2Ω_ profiles extracted along the GeS [100] (blue) and [010] (red) crystallographic directions (bottom) for three different excitation frequencies. The *S*_2Ω_ profiles are averaged as indicated in the respective images with the blue curves offset for visibility. The black continuous lines present fits to the profile using an exponentially decaying sine wave function where applicable.

To experimentally study the excitation of PhPs in GeS within these RBs, we perform polariton interferometry^47^ applying scattering-type scanning near-field optical microscopy (s-SNOM) in combination with a narrowband, tunable FEL.^25^ The experimental setup is sketched in Figure 2a: the pulsed THz radiation produced by the FEL (repetition rate 13 MHz, pulse duration >5 ps) is focused on a metallized atomic force microscopy (AFM) tip that acts as a nanoantenna providing high *k*-vectors along with an enhanced, localized electric field. The polarized tip on top of the GeS flake launches PhPs that propagate away from the tip and are back-reflected at edges of the 224 nm thick flake. The electric field of the back-traveling PhPs is scattered by the same tip into the far field, where it is then detected. By raster scanning the sample (tip is fixed) at a selected incident frequency we obtain a spatial near-field (NF) *S*_2Ω_ image of the polaritons’ interference pattern (see Methods section for details on the setup).^3,4,47^ To ensure that our near-field images are recorded in an area with homogeneous flake thickness and sharp edges, we restrict our s-SNOM measurements to the front-facing flake corner in Figure 2a (equivalent to the bottom right flake corner in Figure 1d).

The near-field image *S*_2Ω_ recorded within spectral area A at an excitation frequency of ν_A_ = 7.02 THz (Figure 2b, upper panel) features two clearly visible, characteristic fringes^4^ with a periodicity of half the polariton wavelength parallel to the horizontal flake edge that are caused by PhP propagation along the [010] direction. In contrast, fringes parallel to the vertical edge are absent, thus indicating a highly anisotropic in-plane propagation of the excited PhPs. In order to determine the experimental complex polariton momentum along the different in-plane crystallographic directions from the near-field intensity image, we extract averaged line profiles along the marked positions starting from the flake edges (as marked in blue and red). Here, the width of the marked profiles represents the number of lines used for averaging that is necessary to suppress the high noise level due to the pulse intensity fluctuations of the THz source. The *S*_2Ω_ profile along the [100] direction (blue curve in Figure 2b, lower panel; offset) indeed does not show features of a propagating polariton, but reflects only the homogeneous dielectric response of the GeS flake. On the other hand, the profile along the [010] direction clearly features the signature of a polaritonic wave pattern, showing three characteristic fringes. By fitting a damped sinusoidal function (black curve) to the data (see Supporting Information Note S4 for details on the fitting procedure and Note S5 for information on noise levels and fitting errors), we identify the PhP momentum to be *k*_*y*,exp_^7.02^ = [(1.4 ± 0.1) + (0.18 ± 0.09)*i*] × 10^4^ cm^–1^ (λ_*y*,exp_^7.02^ ≈ 4.5 μm), meaning a 9.5 times smaller wavelength as compared to the incident THz light.

The second near-field image recorded within spectral area B at a selected excitation of ν_B_ = 7.33 THz (Figure 2c, upper panel) shows two polaritonic fringes along the [010] direction, analogous to the previous case for spectral area A. However, the wavelength of the PhP appears to be significantly smaller than that at ν_A_. Note that the high near-field signal at the vertical edge is attributed to an edge effect caused by scattering of the incident light (the extracted profile along the [100] direction, blue data points in the lower panel of Figure 2c, confirms the high *S*_2Ω_ signal to appear at distances to the edge of *x* < 0, confirming its nonpolaritonic nature). By performing a fitting to the PhP’s signal along the [010] direction, we obtain *k*_*y*,exp_^7.33^ = [(2.44 ± 0.24) + (0.30 ± 0.24)*i*] × 10^4^ cm^–1^ (λ_*y*,exp_^7.33^ ≈ 2.6 μm), indeed exceeding the momentum at ν_A_ (meaning smaller PhP wavelength).

Lastly, the near-field image in Figure 2d is taken at ν_C_ = 8.57 THz, i.e., within spectral area C. It shows polariton-induced fringes parallel to both flake edges. In particular, the fringe spacing parallel to the vertical edge is considerably larger than the fringe spacing parallel to the horizontal edge. Moreover, while up to three distinct fringes are visible decaying along the [100] direction, the fringes decaying along the [010] direction vanish quickly with distance from the flake edge. Accordingly, the respective profiles in Figure 2d (bottom) clearly show features of an exponentially decaying PhP electric field. Through fitting we retrieve the momenta along the [100] and [010] directions to *k*_*x*,exp_^8.57^ = [(3.2 ± 0.2) + (0.3 ± 0.2)*i*] × 10^4^ cm^–1^ (λ_*x*,exp_^8.57^ ≈ 2.0 μm) and *k*_*y*,exp_^8.57^ = [(8.4 ± 0.1) + (1.6 ± 0.8)*i*] × 10^4^ cm^–1^ (λ_*y*,exp_^8.57^ ≈ 0.75 μm), respectively. In this case, the difference in *Re*(*k*) along the two directions is large, which is induced by the unequal in-plane permittivity tensor components.

### PhP Dispersion

**Figure 3:**
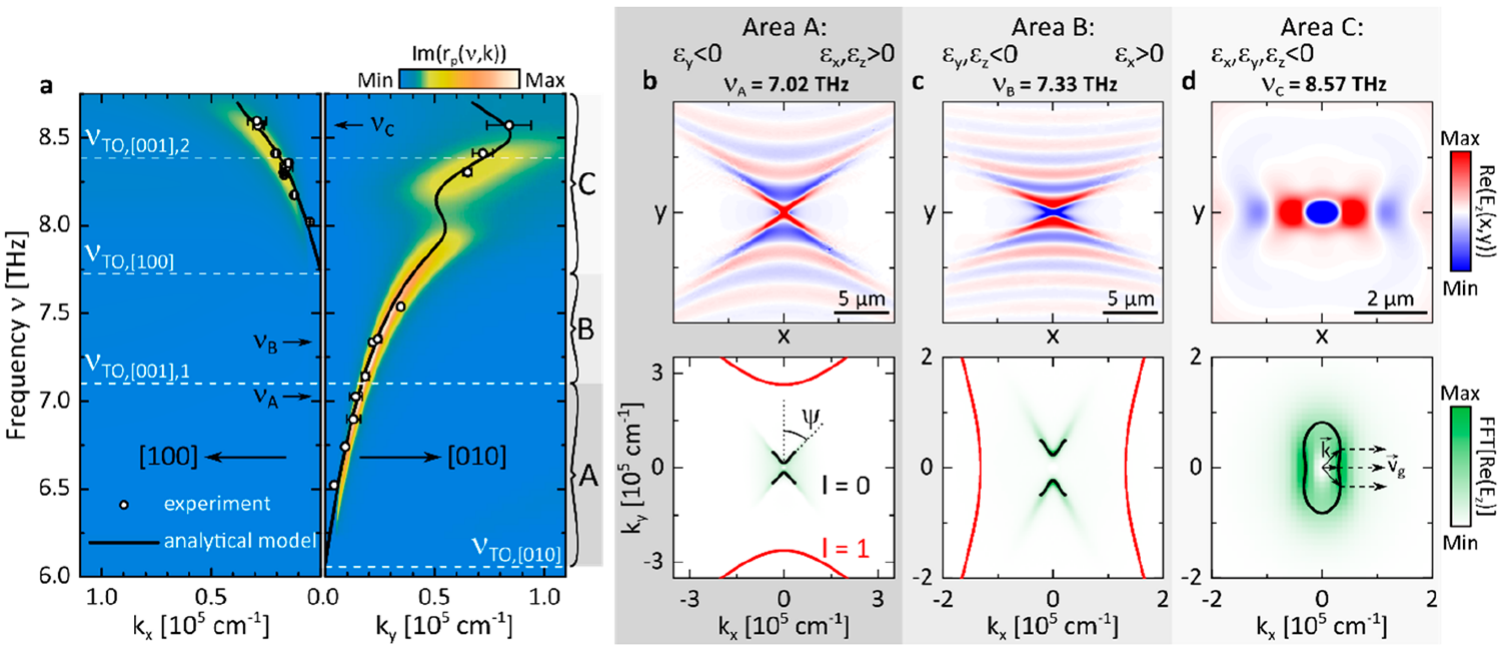
Dispersion and characteristic propagation of PhPs in GeS. (a) Dispersion ν(*k*) along the [100] (left panel) and [010] (right panel) crystal directions for a 224 nm thick GeS slab. The symbols represent the experimental data extracted from near-field profiles, and the black curve corresponds to eq 1 with *l* = 0 and *φ* = 0, π/2. The false-color plot presents the imaginary part of the reflection coefficient *r*_p_(ν, *k*) calculated via the transfer-matrix formalism.^49^ (b, c, d) Numerically simulated PhP field distributions *Re*(*E*_*z*_) (top) and their corresponding *k*-space representation FFT(*Re*[*E*_*z*_]) overlaid with analytically calculated IFC (bottom) for three different frequencies. The full-wave simulations were performed assuming a 224 nm thick GeS slab on top of a silicon substrate. The analytical IFCs (solid lines) given by eq 1 relate to polaritonic modes for both *l* = 0 (black) and *l* = 1 (red). At ν_A_ = 7.02 THz, the PhP dispersion opening angle ψ is illustrated. At ν_C_ = 8.57 THz, the directions of the group velocities (dashed arrows) for selected *k*-vectors (solid arrows) are schematically depicted.

The fundamental PhP dispersion ν(*k*) intrinsic to GeS is obtained experimentally by recording near-field images at various illuminating frequencies in the frequency range ν = 6.0–8.7 THz and fitting the extracted *S*_2Ω_ profiles (symbols in Figure 3a). The left (right) panel relates to PhPs propagating along the [100] ([010]) direction, starting at the TO frequency, ν_TO_, where the permittivity becomes negative along the respective in-plane direction. The black curves present the PhP wavevectors *Re*[*k*(ν)] calculated using the equation for the polariton in-plane wavevector *k*^2^ = *k*_∥_^2^ = *k*_*x*_^2^ + *k*_*y*_^2^ recently derived for a biaxial slab:^13,48^
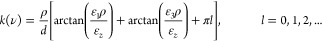
1with the slab thickness *d*, the permittivity of the superstrate (substrate) *ε*_1_ (*ε*_3_), the mode quantization index *l*, the GeS permittivity tensor diagonal elements *ε*_*x*_, *ε*_*y*_, *ε*_*z*_, using, where *φ* is the angle between *k* and the *x*-axis. Notably, the analytical curves are in excellent agreement with our experiment (a minor adjustment of the GeS permittivity was done for RB_*x*_, see Supporting Information Note S3). Moreover, we evaluated the reflectivity *r*_p_(ν, *k*) of the layered air/GeS/Si system via the transfer matrix formalism.^49^ The resulting Im[*r*_p_(ν, *k*)] contains information on both the polariton dispersion and damping, with the positions of the maxima yielding the PhP dispersion and their width being directly related to their damping Im(*k*). We find that the Im[*r*_p_(ν, *k*)] (false-color plot in Figure 3a) matches excellently the experimental and analytical data, thus unambiguously supporting our observations.

Along the [100] direction we find a phonon polariton branch emerging on the dispersion plot above ν_TO,[100]_ = 7.74 THz (left panel in Figure 3a): the momentum *Re*(*k*_*x*_) increases with frequency ν up to *Re*(*k*_*x*_) = 0.4 × 10^5^ cm^–1^ (i.e., with a positive group velocity) with the relatively large width of the Im[*r*_p_(ν, *k*)] peak, indicating considerable damping. Along the [010] direction, in the frequency range ν = 6.06–7.9 THz, we comparably observe the polariton momentum *Re*(*k*_*y*_) to increase with frequency up to *Re*(*k*_*y*_) = 0.55 × 10^5^ cm^–1^, accompanied by a smaller damping as compared to the polariton branch along the [100] direction. However, at higher frequencies ν = 7.9–8.2 THz and similarly for ν > 8.5 THz, the dispersion becomes much more intricate due to two separate areas of negative group velocity (anomalous dispersion) emerging along the [010] direction: the previously monotonically increasing dispersion bends back, with the derivative dν(*k*)/d*k* becoming negative. As seen in the reflectivity Im[*r*_p_(ν, *k*_*x*_)], this effect is accompanied by a substantial polariton damping that renders it challenging to be observed in the experiment. For this reason, a supporting full-wave theoretical investigation has been carried out as stated in a later section of this work, while a detailed discussion is given in Note S6 of the Supporting Information. Anticipating the results, we find the anomalous PhP dispersion in GeS (i) to be induced by the *z*-phonons spectrally overlapping with the in-plane reststrahlen bands and (ii) to be mediated by the substrate. Near the low-frequency limit of the second back-bending regime, we measure the highest momenta of *Re*(*k*_*y*_) = 0.84 × 10^5^ cm^–1^ (λ_*y*_ = 0.75 μm) at the frequency ν = 8.57 THz.

In a nutshell, the highly anisotropic permittivity of GeS governed by overlapping degenerate optical phonon modes in a narrow spectral regime introduces an exotic in-plane PhP dispersion. The latter features several back-bending effects and three characteristic areas A, B, and C, with different polariton modes that will be discussed in the following.

### Simulated PhP Propagation

In order to explore in depth the PhP in-plane propagation within the three different spectral areas defined by the GeS permittivity (Figure 1e) and reflected by the PhP dispersion (Figure 3a), we carried out full-wave electromagnetic simulations at the representative excitation frequencies ν_A_, ν_B_, and ν_C_ (corresponding to the experimental data in Figure 2b–d). More specifically, we simulate the electromagnetic fields generated by a vertical point dipole above a GeS slab, in analogy to an illuminated AFM tip. The presented component *Re*(*E*_*z*_) is directly linked to the experiment, as it provides a valid numerical description of the signals measured in s-SNOM^50^ (see Methods section and Note S7 in the Supporting Information).

#### Area A, 6.06–7.1 THz (ν_TO,[010]_–ν_TO,[001],1_)

The simulated *Re*[*E*_*z*_(*x*, *y*)] image for the frequency ν_A_ = 7.02 THz within RB_*y*_ shown in Figure 3b (color plot, top panel) reveals an unusual polaritonic field distribution: launched by the exciting dipole located at the center of the graph, a polariton propagates within a sector centered in the *y* (=[010])-direction featuring hyperbolic wavefronts. Notably, no PhPs with wavevectors along the *x* (=[100])-direction are allowed, while the PhPs propagating along the *y*-direction have momenta *k*_*y*,sim_^7.02^ = [(1.78+ 0.18*i*) × 10^4^ cm^–1^ (λ_*y*,sim_^7.02^ ≈ 3.35 μm). The bottom image of Figure 3b depicts the corresponding PhP representation in momentum space. To that end, the green color plot presents the fast Fourier transform (FFT) of the numerical real-space image above: The isofrequency curves (IFCs, sections of the dispersion surface for a constant frequency) of polaritons present hyperbolas with their major axes aligned along the *k*_*y*_-direction and an opening angle of ψ_sim_^7.02^ ≈ 41°. In addition, we obtained the analytical IFCs applying eq 1 for propagating PhP modes [*Re*(*k*) > Im(*k*)] with quantization indices *l* = 0 (black curve) and *l* = 1 (red curve): both curves hold a hyperbolic shape with similar orientation, with the *l* = 0 mode matching the simulation and the *l* = 1 mode exhibiting higher in-plane momenta. In particular, the zero-order PhP momentum along *k*_*y*_-direction anticipated by the IFC amounts to *k*_*y*,calc_^7.02^ = (1.49 + 0.15*i*) × 10^4^ cm^–1^ (λ_*y*,calc_^7.02^ ≈ 4.5 μm), matching excellently the experimentally obtained value. However, signatures of the calculated higher order mode are lacking in both the simulated field distribution and the experimental near-field images. Lastly, we calculate the opening angle of the hyperbola, defined by^31^, yielding ψ_calc_^7.02^ = 39° at the frequency ν_A_, which is in good agreement with the value obtained from the simulation.

#### Area B, 7.1–7.74 THz (ν_TO,[001],1_–ν_TO,[100]_)

At ν_B_ = 7.33 THz the simulated in-plane field distribution in the top panel of Figure 3c likewise shows propagating PhPs featuring characteristic hyperbolic wavefronts. A direct comparison to the field distribution at ν_A_ (Figure 3b) reveals a higher in-plane momentum (i.e., shorter wavelength) alongside an increased angular spread (relating to a decrease in the PhP opening angle ψ). More precisely, the numerical simulation yields a complex momentum along the *y*-direction of *k*_*y*,sim_^7.33^ = (2.42 + 0.26*i*) × 10^4^ cm^–1^ (λ_*y*,sim_^7.33^ ≈ 2.60 μm). Furthermore, note that the change in sign of the out-of-plane component *Re*(*ε*_*z*_) induces an offset of π to the PhP phase as compared to that in area A. Accordingly, the FFT of the lateral field distribution in the bottom panel of Figure 3c describes open hyperbolas with their major axes aligned parallel to the *k*_*y*_-direction and an opening angle of ψ_sim_^7.33^ ≈ 30°. The PhPs’ hyperbolic character is further supported by their analytically calculated IFC matching the findings from the simulation. Being in good agreement with the FFT[*Re*(*E*_*z*_)], the black curve with mode index *l* = 0 yields a PhP momentum of *k*_*y*,cal_^7.33^ = (2.3 + 0.23*i*) × 10^4^ cm^–1^ (λ_*y*,cal_^7.33^ ≈ 2.73 μm), matching the value extracted from the experiment. Furthermore, the analytical opening angle amounts to ψ_calc_^7.33^ ≈ 28°, in agreement with our simulations. Intriguingly, the change of sign in *Re*(*ε*_*z*_) leads to a rotation of the IFC for *l* = 1 by π/2, as anticipated from theory.^48^ While we are unable to resolve this rotation experimentally, a more detailed theoretical discussion of the hyperbolic higher order PhP mode residing in the GeS volume and its rotation at the frequency of the zero-crossing of *Re*(*ε*_*z*_) at ν_TO,[001],1_ is presented in the Supporting Information Note S8.

#### Area C, 7.74–9.47 THz (ν_TO,[100]_–ν_LO,[010]_)

The simulated field distribution *Re*(*E*_*z*_) for a third frequency ν_C_ = 8.57 THz that is located within the overlap between the GeS in-plane reststrahlen bands RB_*x*_ and RB_*y*_ is presented in Figure 3d. Here, as the real parts of the corresponding permittivities *Re*(ε_*x*_) and *Re*(ε_*y*_) are both negative and, moreover, show a high degree of anisotropy (*Re*(ε_*x*_)/*Re*(ε_*y*_) = 3.4), the spatial distribution of *Re*(*E*_*z*_) reveals an elliptically propagating polariton with largely different wavevectors along the in-plane crystal directions. Interestingly, the wavefronts along the *x*-direction hold a faint hyperbolic shape, as compared to the convex shape along the *y*-direction. The simulation predicts the [100] crystal axis to host low-loss polaritons with *k*_*y*,sim_^8.57^ = (3.31 + 0.93*i*) × 10^4^ cm^–1^ (λ_*x*,sim_^8.57^ ≈ 1.90 μm). In contrast, polaritons propagating along the [010] direction have a nearly 2-fold increased momentum *k*_*y*,sim_^8.57^ = (6.49 + 8.2*i*) × 10^4^ cm^–1^ (λ_*y*,sim_^8.57^ ≈ 0.97 μm), accompanied by higher losses. Note that the phase of the field at the dipole position is similar to that at ν_B_ (blue color), which is consistent with the same (negative) sign of *Re*(*ε*_*z*_). The FFT of the simulated field distribution is presented in the bottom panel of Figure 3d, where we find a peculiar distribution of spatial PhP momentum. The latter holds an elliptical shape with higher momenta and broadened distribution (corresponding to higher damping) along *k*_*y*_ compared to *k*_*x*_. The overlaid IFC (black curve) matches well the simulated data, even highlighting further an intricate feature along *k*_*x*_: in fact, the IFC is not of an ideal elliptical shape, but holds some hyperbolic features, fitting to the observations from the *Re*[*E*_*z*_(*x*, *y*)] distribution. The PhP momenta anticipated from the IFC amount to *k*_*x*,cal_^8.57^ = (3.0 + 1.1*i*) × 10^4^ cm^–1^ and *k*_*y*,cal_^8.57^ = (8.2 + 7.0*i*) × 10^4^ cm^–1^ (λ_*x*,cal_^8.57^ = 2.09 μm and λ_*y*,cal_^8.57^ = 0.77 μm), respectively, again in good agreement with the simulations. Note that no propagating modes with quantization index *l* > 0 exist, since the PhP holds a surface character (having mostly imaginary momentum across the surfaces of the slab) due to the purely negative GeS permittivity in this spectral range.^48^ Remarkably, the “propeller”-shaped IFC together with the high PhP damping along the [010] direction leads to a tantalizing, apparent canalization of the PhP. As illustrated by the parallel orientation of the group velocities *v*_g_ depicted for selected in-plane PhP momenta *k* (Figure 3d, bottom) and, moreover, visible in the *Re*[*E*_*z*_(*x*, *y*)] image (Figure 3d, top), the PhPs propagate with neither perfectly hyperbolic nor elliptical but rather planar wavefronts. The observed propagation of PhP in GeS closely resembles the canalization of PhPs recently found in twisted slabs of α-MoO_3._^30−33^ Similar effects have been studied theoretically in plasmonic and phononic metamaterials^19,51,52^ although, to our knowledge, have not been observed in a single layer of a natural material, yet. A detailed theoretical analysis of this canalized polariton propagation in GeS is presented in the last section of this work and, specifically for ν_C_ = 8.57 THz, in Note S9 in the Supporting Information. Direct experimental observation of this phenomenon would require an antenna on the sample for PhP excitation, instead of launching it via the s-SNOM tip. Hence, a dedicated, frequency-dependent experimental study of this canalization effect will be presented in a future work.

### Properties of the GeS THz Polaritons

Ultimately, to thoroughly characterize the PhPs in GeS and, thereby, paving the way towards applications, we determine the key GeS polaritonic properties, which are the quality factor *Q*, lifetime τ, and light confinement β. We compare these values obtained from our experiment to the analytical model, and contrast them to recent PhP-hosting materials.

**Figure 4:**
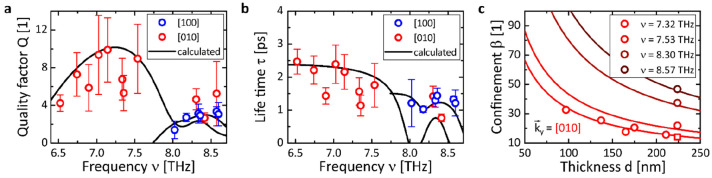
Characteristic properties of PhPs in GeS. The individual data points are extracted from our experiment, while the solid lines are calculated from eq 1. (a) Quality factors as a function of frequency for PhPs propagating along the [100] (blue) and [010] (red) crystal directions. (b) Frequency-dependent polariton lifetime along the two in-plane crystal directions. (c) Thickness dependence of the polariton field confinement at four different frequencies within RB_*y*_. The experimental data taken at ν = 7.32 THz for a set of different GeS flakes follow the well-known ∼1/*d* behavior. The squared data point was taken at a negligibly different frequency, ν_B_ = 7.33 THz, on the *d* = 224 nm thick flake.

The quality factor *Q* = *Re*(*k*)/Im(*k*) presents a practical figure of merit that (in real space) relates the polariton’s wavelength to its decay length.^6^ For GeS, in Figure 4a we find quality factors of up to *Q* = 10 in RB_*y*_ and *Q* = 3 in RB_*x*_ along the [010] and [100] directions, respectively. The black curves are obtained directly from eq 1 and describe well the experimental data. The lower values of the experimentally extracted quality factors (and, likewise, lifetimes in Figure 4b) at 6.9, 7.33, and 7.35 THz as compared to the modeled ones can be ascribed to an increased bandwidth of the exciting FEL pulse that leads to an artificially increased polariton damping (more detailed explanation given in the Supporting Information Note S10). Note that the quality factor drops at the in-plane LO and TO frequencies as well as in the regions of the back bending in the dispersion. Overall, the quality factors resemble those reported for α-MoO_3_ (*Q* ≈ 7–12),^25^ are 2 times smaller than for naturally abundant hBN (*Q* ≈ 20),^20^ and are about 3 times higher than in α-V_2_O_5_ (*Q* = 2.5).^5^

The GeS PhPs lifetime τ = [*v*_g_*Im*(*k*)]^−1^ (with the group velocity *v*_g_ = 2π*c* dω/d*k* [ω = λ_0_^–1^ denoting the wavenumber], presented in Note S11 of the Supporting Information) lies in the picosecond range (Figure 4b) as anticipated for low-loss PhPs.^53^ Within RB_*y*_, lifetimes of up to τ_[010]_ = 2.3 ps can be found, while the lifetimes in RB_*x*_ are considerably smaller with τ_[100]_ < 1.4 ps. The lifetimes are thus comparable with those reported for PhPs in hBN (<2 ps),^20^ but shorter than in case of PhPs in α-MoO_3_ (2–8 ps)^4,25^ and α-V_2_O_5_ (3–6 ps).^5^ Note that in the same way as for the quality factor a higher excitation bandwidth can artificially decrease the extracted experimental lifetime (see Supporting Information Note S10).

Moreover, the large errors in the determination of lifetime as well as quality factor are related to the low signal-to-noise ratio within the experiment (typical error margins are Δ*Re*(*k*)/*Re*(*k*) = 10% and ΔIm(*k*)/Im(*k*) = 25%, resulting in Δ*Q*/*Q* = 35% and Δτ/τ = 25%; see Note S5 in the Supporting Information), which is consistent with our simulations: for GeS, we find a smaller overall polaritonic field *Re*(*E*_*z*_) for a given driving field strength as compared to α-MoO_3_ and hBN, for example.

In addition, it is important to note that following the common definition of the lifetime (propagation length *L* = Im(*k*)^−1^ divided by the group velocity *v*_g_) can erroneously lead to negative values (in the regions of anomalous dispersion, where d*ν*/d*k* < 0) as, for instance, in the dispersion curve along the [010] direction in Figure 3a. Therefore, in the anomalous dispersion region, one has to use a different (more general) determination of the lifetime, based on the eigenmode analysis in the space of a complex frequency and a real wavevector.^10^ Finally, we calculate the thickness-dependent light confinement β = *k*/*k*_0_ = λ_0_/λ (that is, the ratio of the incident, free-space wavelength λ_0_ with respect to the polariton wavelength λ = 2π/*k*) at different frequencies in RB_*y*_. As presented in Figure 4c, the experimental values of β follow very well the ∼1/*d* dependence anticipated from eq 1 (solid curves). We find in our experiment the highest field confinement of β = 47 in the 224 nm thick GeS flake at ν_C_ = 8.57 THz, whereas considerably larger values are expected for thinner flakes.

### Dispersion Back-Bending and Polariton Canalization

Lastly, we elaborate by theoretical means the two unconventional phonon-polaritonic effects in GeS specific to spectral area C: (i) the dispersion back bending found using the analytical models [eq 1 and the TM formalism, Figure 3a] and (ii) the PhP canalization observed in the full-wave simulation (Figure 3d).

#### i PhP Dispersion Back-Bending

In general, back bending of a polaritonic dispersion is well-known and can take place due to several physical reasons. First, it may occur in the vicinity of the spectral range where the dielectric permittivity becomes negative.^54,55^ In this case, the bending appears near the light line and the polariton branch emerging in the area with *Re*(ε) > 0, which lacks interface confinement. Second, polaritons coupling to external excitations (such as phonons of the substrate^56^ or nearby molecular resonances^10^) have been reported to induce back bending to an otherwise monotonic polariton dispersion. Finally, an anomalous polariton dispersion can be induced by the PhPs coupling to intrinsic phonons, which was previously observed in α-MoO_3_.^57^ For the latter, the in-plane elliptical PhP mode (that is caused by a negative permittivity in the vdW stacking direction) couples to a weak phonon along the [100] direction (located at ω_TO_ = 998.7 cm^–1^), resulting in the dispersion back bending precisely along that direction.

**Figure 5:**
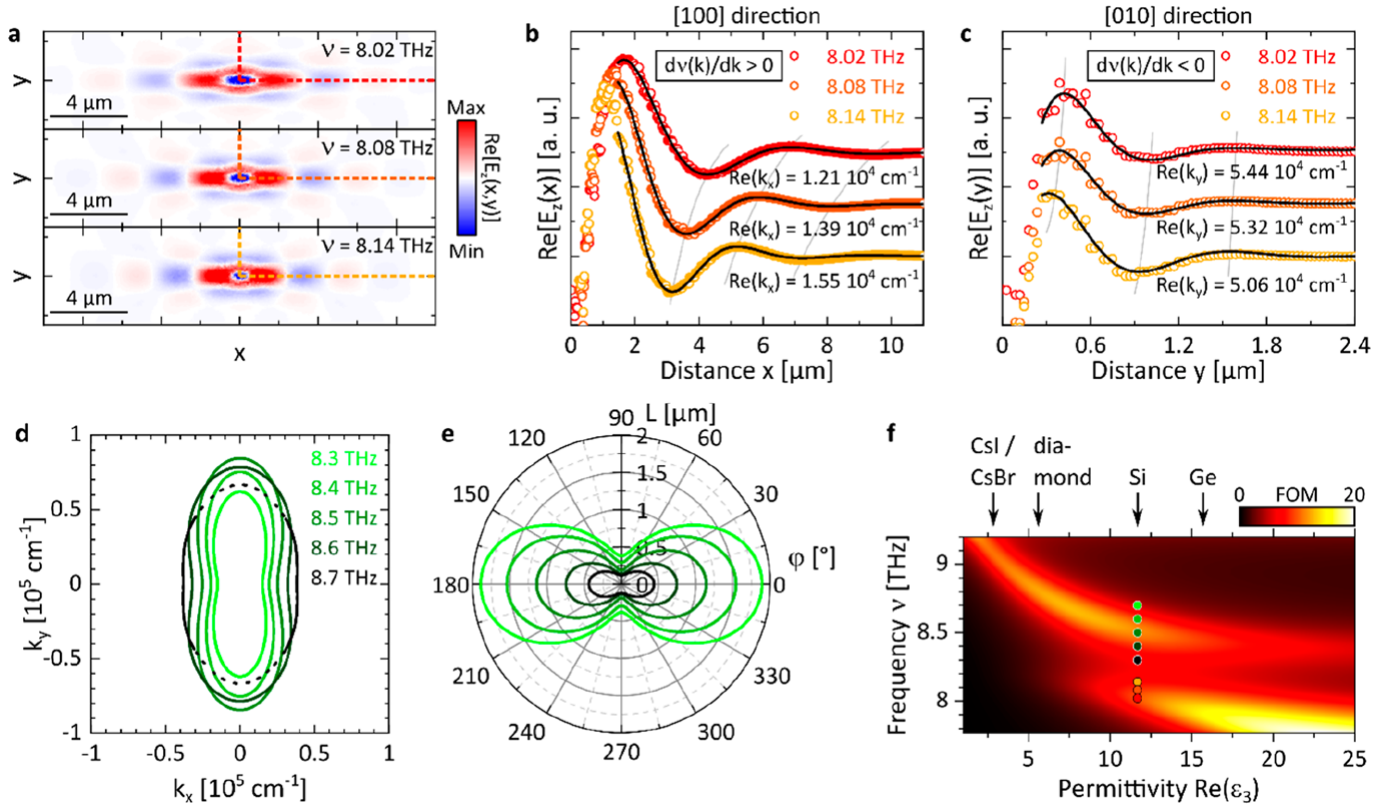
Dispersion back bending and PhP canalization in GeS. (a) Simulated in-plane field distributions *Re*[*E*_*z*_(*x*, *y*)] at frequencies within the lower back-bending area of ν(*k*_*y*_) in Figure 3a. The dashed lines in the 20 × 5 μm^2^ areas mark the positions where the profiles in (b, c) were extracted. (b, c) PhP field profiles fitted using decaying sine functions to obtain the momentum Re(*k*) along the *x-* and *y*-direction, respectively (curves are offset for the sake of visibility). Along the *x*-direction the PhP dispersion ν(*k*) has a positive slope dν(*k*)/d*k*, whereas it is negative along the *y*-direction. (d) Isofrequency curves calculated after eq 1 in the frequency regime, where canalized PhP propagation was found in Figure 3d. The dashed lines refer to an overdamped propagation. (e) Angle-resolved in-plane PhP propagation length *L* = Im(*k*)^*–*1^ at the frequencies in (d) sharing the same color code. (f) Canalization figure of merit as a function of the substrate’s permittivity *Re*(ε_3_) calculated within the RB_*x*_ frequency range. The permittivity of typical THz materials is indicated at the top. The position of the field distributions in (a) and the graphs in (d, e) are marked by colored symbols.

In order to first substantiate the indicated dispersion back bending in α-GeS along the [010] direction (see Figure 3a, right panel), specifically at around ν = 8.1 THz, where experimental evidence is lacking, additional full-wave simulated PhP field distributions *Re*[*E*_*z*_(*x*, *y*)] are provided in Figure 5a. For the three excitation frequencies shown within the back-bending regime, the in-plane polariton propagation greatly differs along the [100] and [010] directions: the former is characterized by a longer wavelength that decreases with frequency and considerably longer propagation length, whereas the latter features short wavelengths and substantial damping. To quantify the frequency-dependent behavior, *Re*(*E*_*z*_) profiles were extracted along the *x-* and *y*-direction (Figure 5b,c) and fitted using a decaying sine function to obtain the PhP momentum. Whereas for the [100] direction (Figure 5b), *Re*(*k*_*x*_) increases with frequency corresponding to a normal dispersion with positive group velocity, it decreases with frequency for the [010] direction (Figure 5c), which is in line with the expected dispersion back bending with negative group velocity. Moreover, the PhP damping in the *y*-direction is very high (*Q*_[010]_^sim^ ≈ 2.0), thus complicating its experimental observation: even with improved, low-noise laser sources, we would anticipate that only the first PhP field oscillation could be visible in the near-field image.

Note that the origin of the anomalous PhP dispersion in GeS is different from the aforementioned phonon-polariton-to-phonon coupling (both in-plane) in α-MoO_3_, as extensively demonstrated in the Supporting Information Note S6: in GeS, the broad spectral overlap of the in-plane reststrahlen bands with the two *z*-phonon modes induces the back bending of the PhP dispersion, notably without a coupling effect that would be pronounced at the TO *z*-phonon frequencies. In particular, we find the strong (weak) *z*-phonon with *ν*_TO,[001],1_ = 7.1 THz (ν_TO,[001],2_ = 8.4 THz) to induce the back bending around ν = 8.5 THz (8.0 THz). The significant difference between the progression of the PhP dispersion along the [010] and [100] direction can be attributed to the largely different  term (*i = x*, *y*) in eq 1 suppressing the back-bending effect along the [100] direction. Moreover, it is the substrate-related tan^–1^(ε_3_ρ/*ε*_*z*_) term that introduces the back-bending effect through strong modulation of the wave reflection at the GeS/Si interface due to the evolution of the complex *ε*_*z*_(ν) (see Figures S5 and S7 in the Supporting Information). Consequently, the magnitude and spectral location of the dispersion back bending (along both in-plane directions) is to a large extent substrate dependent, with a suspended flake notably showing no anomalous dispersion.

#### ii PhP Canalization

In general, a canalized (highly directional) propagation of polaritons is characterized by an IFC featuring parallel straight lines in momentum space (i.e., the group velocities for a large continuum of *k* vectors are parallel). In the spectral regions where the dispersion of PhPs in a GeS slab exhibits back bending, significantly elongated elliptical IFCs appear (Figure 3d), so the propagation of PhPs is highly directional. All the IFCs calculated around the frequency of 8.57 THz according to eq 1 show such a characteristic shape (Figure 5d). Notably, the highest momentum *Re*(*k*_*y*_) is found right before the back-bending regime at 8.5 THz, after which the values decrease with frequency. The elongation of IFCs is also accompanied by a highly anisotropic damping, as illustrated in Figure 5e, where the directional and frequency-dependent PhP propagation length *L*(φ) holds an elongated shape (similar to that of the IFCs) with the values generally decreasing with frequency. In particular, the ratio *L*_*x*_/*L*_*y*_ has its maximum (i.e., highest anisotropy) at 8.5 THz (*L*_*x*_/*L*_*y*_ = 6.17), hence greatly emphasizing the directional PhP propagation along the [100] direction.

For the purpose of assessing the canalized propagation of the GeS PhP at a given excitation frequency, we introduce here a practical figure of merit (FOM) as
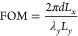
with *d* the thickness of the flake. This definition includes both the ratio of propagation lengths *L*_*x*_/*L*_*y*_ = Im(*k*_*y*_)/Im(*k*_*x*_) and the elongated shape of the IFC that results in the parallel alignment of the group velocities. We note that the literal expression for the elongation of the ICF ellipse, λ_*x*_/λ_*y*_, diverges near *ν*_TO,[100]_ = 7.74 THz, and hence we use the term 2π*d*/λ_*y*_, which represent a similar measure for such elongation. Further note that the introduced FOM is useful specifically for PhP canalization in GeS along the [100] direction. The FOM presented in Figure 5f for a silicon substrate (ε_3_ = ε_Si_) features two distinct maxima, one located at 8.5 THz and a second, weaker one at 8.05 THz. The first maximum is well in line with the PhP canalization that is apparent in Figure 3d at 8.57 THz, whereas the second relates to the field distributions in Figure 5a: here, the PhP propagation shows similar characteristics, albeit not as pronounced as at 8.57 THz.

We would like to highlight that the PhP canalization in GeS slabs appears in the spectral areas of anomalous dispersion. Indeed, the back-bending areas manifest a strong damping along the [010] direction with high *Re*(*k*_*y*_), while in the orthogonal axis [100], *Re*(*k*_*x*_) and Im(*k*_*x*_) are virtually unaffected. Accordingly, such anisotropy in damping leads to the best canalization FOM near the low-frequency limit of the anomalous dispersion regimes. Interestingly, as the spectral position of the back-bending areas are highly dependent on the substrate’s permittivity, the possibilities for tuning the canalization regime become apparent, as illustrated in Figure 5f. With decreasing permittivity of the substrate *Re*(ε_3_), with respect to the value of Si, the upper canalization regime (around 8.5 THz) shifts toward higher frequencies, whereas the lower regime (around 8.05 THz) quickly vanishes. An increase of the substrate’s permittivity causes the canalization FOM in the upper regime to decrease, while the FOM in the lower regime greatly increases and shifts toward lower frequencies. This behavior of the canalization is based on the substrate dependence of the back-bending areas (see Figure S7 in the Supporting Information): The evolution of the upper canalization regime with increasing *Re*(ε_3_) is due to the shift of the high-frequency back-bending area along the [010] direction down to lower frequencies, with the canalization disappearing due to an overlap of the back-bending areas along the [010] and [100] direction (i.e., in-plane PhP highly damped). On the other hand, the lower canalization regime occurs when the low-frequency back-bending area along the [010] direction emerges, which shifts toward the edge of the spectral area C, ν_TO,[100]_ (only one back-bending area exists along the [100] direction for 1 < *Re*(ε_3_) < 25).

From a practical perspective, our results show how to achieve canalization of PhPs in single slabs of natural crystals, an effect that up to now was just observed by fabricating twisted stacks of polaritonic materials. Notably, the bandwidth of the canalization effect (fwhm ≈ 0.2–0.3 THz, extracted from Figure 5f) in GeS exceeds that of the extremely narrowband PhPs in twisted slabs of α-MoO_3_, although the propagation length of the latter is longer.

## Conclusion

In conclusion, we extensively explored by means of experimental and theoretical methods the properties of THz phonon polaritons in thin slabs of the highly anisotropic vdW semiconductor α-GeS. We revealed strongly confined and in-plane anisotropic polaritonic modes at frequencies ranging from 6 to 9 THz. The characterized low-loss PhPs feature long lifetimes (τ > 2 ps), together with an excellent figure of merit (*Q*_max_ = 10) and THz lightwave confinement (β > 45). Moreover, the anomalous dispersion and the anticipated natural canalization effect of PhPs in this material are of particular interest, both originating from the interplay of the highly directional RBs and being tunable via the substrate material selection. For these reasons, GeS promises to become a feasible, versatile platform for THz light confinement and manipulation. Moreover, we envision that the work presented here will inspire further research on THz PhPs: while on the one hand, the material family presents a toolbox for THz PhP engineering (for example via stacking and twisting), on the other hand, GeS as a semiconductor holds the promise of potentially tuning the PhPs via electrostatic gating (i.e., PhP–electron interaction). Moreover, the possibility of direct control of the charge carrier concentration may enable the study of plasmon–phonon coupling with the goal to actively control the anisotropic polaritons. Lastly, the large thermoelectric effect motivates investigation of the thermoelectric properties of the PhPs that could potentially be probed via photocurrent nanoscopy.^58^ To that end, the scarcity of suitable THz sources currently presents the only limitation.

## Methods

### Scattering-Type Scanning Near-Field Optical Microscopy

The near-field images were recorded applying a (modified) commercial near-field microscope (Neaspec GmbH, Germany) integrated with the free-electron laser located at Helmholtz-Zentrum Dresden-Rossendorf (HZDR), Germany. By illuminating the oscillating (Ω = 250 kHz), metallized s-SNOM tip in the vicinity of the sample surface, the excited tip acts as an antenna, providing a strong, localized electric field at its apex. The confined field interacts with the sample volume, and hence, its local optical response becomes imprinted in the backscattered signal *S*. In order to separate the sample’s near-field optical response from the dominant far-field background, the nonlinear distance dependence of the NF contribution (as compared to the linear dependence of the far field) is exploited:^59^ by employing a lock-in amplifier we obtain individual components of the scattered signal at multiples of the cantilever oscillation frequency *S*_*n*Ω_ (*n* = 1, 2, 3, ...) and find effective background suppression in the components with^60^*n* ≥ 2. Throughout this work, a self-homodyne detection scheme was applied and the backscattered optical signal was demodulated at *n* = 2. The optical signal at the frequencies ν = 6–9 THz was recorded using a gallium-doped germanium photoconductive detector by QMC Instruments Ltd., UK.

### Free-Electron Laser

Light sources in the THz spectral regime that are suitable for s-SNOM application currently present a major limitation to near-field optical investigation of collective excitations in condensed matter physics. Established table-top solutions such as gas lasers or quantum cascade lasers are restricted by either the small range of accessible frequencies or the lack of sufficient spectral power density.^61^ In contrast, light emission of relativistic electrons can be exploited in large-scale facilities (namely, synchrotrons or free-electron lasers) to provide either broadband (in the first case) or continuously tunable, narrowband THz radiation (in the latter case). While synchrotron infrared nanospectroscopy currently is operational at frequencies down to >9.6 THz,^26,62^ FELs in particular have been successfully applied in s-SNOM in the range 1.3–30 THz.^25,63^

In this work, we apply the free-electron laser FELBE at the ELBE Center for High Power Radiation Sources at HZDR, Germany, capable of generating coherent THz radiation over the spectral range of 1.2–60 THz with a repetition rate of 13 MHz. Particularly the U100 FEL oscillator provides the required brightness to launch and detect PhPs in the 6–9 THz spectral regime. The spectral bandwidth of individual pulses was minimized by slightly detuning the cavity, resulting in values of about 0.5–0.9%_fwhm_ and transform-limited pulse durations of >5 ps. The implied pulse spectral diagnostic was performed applying a Czerny–Turner-type scanning grating spectrometer (Princeton Instruments SP-300i).

### Full-Wave Numerical Simulations

The structures were modeled as biaxial GeS slabs on top of high-resistivity float-zone Si substrates. In s-SNOM experiments the tip acts as an optical antenna that converts the incident light into a strongly confined near-field below the tip apex, providing the necessary momentum to excite PhPs. However, owing to the complex near-field interaction between the tip and the sample, numerical quantitative studies of s-SNOM experiments meet substantial difficulties in simulating near-field images.^64^ To overcome these difficulties, we approximate the tip by a dipole source (with a constant dipole moment),^50^ in contrast to the usual dipole model, in which the effective dipole moment is given by the product of the exciting electric field and the polarizability of a sphere.^65^ We assume that the polarizability of the dipole is weakly affected by the PhPs excited in the GeS slab, and their back-action onto the tip can be thus neglected. Therefore, we place a vertically oriented point electric dipole source on top of the GeS slab and calculate the amplitude of the near field, |*E*_*z*_|, above the GeS/Si structure, where PhPs propagate. Our simulated images (using Comsol Multiphysics) are in good agreement with our experimental results (see Figure 3), which lets us conclude that the calculated field between the dipole and the GeS flake, *E*_*z*_, provides a valid numerical description of the signals measured by s-SNOM.

## Supporting Information Available

Details on sample preparation and precharacterization (Notes S1 to S3), the near-field optical data analysis (Note S4), the noise figure in the experiment (Note S5), the dispersion back bending (Note S6), three-dimensional representation of the PhP field distributions (Note S7), the rotation of the *l* = 1 mode (Note S8), the PhP canalization (Note S9), the impact of excitation bandwidth on the PhP properties (Note S10), the PhP group velocity (Note S11), and related supplementary references (PDF)
