# Supplementary material for: Germanium Monosulfide as a Natural Platform for Highly Anisotropic THz Polaritons
Source: ACS Nano. 2022 Nov 29;16(12):20174–85. doi: 10.1021/acsnano.2c05376 (PMC9799068; doi:10.1021/acsnano.2c05376)
Supplement: Supplementary file 1 — nn2c05376_si_001.pdf [file nn2c05376_si_001.pdf]

Supporting Information

Germanium monosulfide as a natural platform for highly anisotropic THz polaritons

Tobias Nörenberg [†,‡,§], *Gonzalo Álvarez-Pérez* [‖,#], *Maximilian Obst* [†], *Lukas Wehmeier* [†,‡,‖‖], *Franz Hempel* [†,††], *J. Michael Klopf* [§], *Alexey Y. Nikitin* [‡‡,§§], *Susanne C. Kehr* [†,*], *Lukas M. Eng* [†,‡,††], *Pablo Alonso-González* [‖,#,*], *and Thales V. A. G. de Oliveira* [§,†,‡*]

[†] Institut für Angewandte Physik, Technische Universität Dresden, Dresden 01187, Germany;

[‡] Würzburg-Dresden Cluster of Excellence - EXC 2147 (ct.qmat), Dresden 01062, Germany;

[§] Institute of Radiation Physics, Helmholtz-Zentrum Dresden-Rossendorf, Dresden 01328, Germany;

[‖] Department of Physics, University of Oviedo, Oviedo 33006, Spain;

[#] Center of Research on Nanomaterials and Nanotechnology CINN (CSIC–Universidad de Oviedo), El Entrego 33940, Spain;

[††] Collaborative Research Center 1415, Technische Universität Dresden, Dresden 01069, Germany;

[‡‡] Donostia International Physics Center (DIPC), Donostia-San Sebastián 20018, Spain;

[§§] IKERBASQUE, Basque Foundation for Science, Bilbao 48013, Spain;

[‖‖] Present Address: Brookhaven National Laboratory, National Synchrotron Light Source II, Upton, NY, USA;

Table of Contents

- **Note S1: Sample preparation**
- **Note S2: Polarization-resolved Raman spectroscopy**
- **Note S3: GeS THz permittivity**
- **Note S4: PhP fitting procedure**
- **Note S5: Noise Analysis in FEL-based polariton interferometry**
- **Note S6: Origin of anomalous dispersion**
- **Note S7: 3D representation of the PhP field distribution**
- **Note S8: 90° Rotation of the l = 1 mode at ν = 7.1 THz**
- **Note S9: Natural canalization of PhPs in single slabs of GeS at THz frequencies**
- **Note S10: Impact of the FEL spectral bandwidth on PhP properties**
- **Note S11: PhP group velocity v_g**
- **References**

Note S1: Sample preparation

The commercially available (2Dsemiconductors Inc., USA) semiconducting α-GeS crystal used in this work was grown applying the flux-zone technique and exhibits a purity of >99.9995%. The (bulk) crystal holds an orthorhombic crystal structure (a = 4.29 Å, b = 3.64 Å, c = 10.42 Å; α, β, γ = 90°) with an electric bandgap of 1.65 eV (see **Figure S1**a-d). The studied GeS-flakes (compare Figure S1e,f) were prepared via adhesive tape exfoliation and deposited onto a double-side polished high-resistivity float-zone silicon substrate (HRFZ Si, Tydex LCC, Russia). The substrate was thoroughly cleaned with deionized water, acetone and isopropyl alcohol. Following a drying process (nitrogen air blow), the substrate surface was plasma treated (O_2, 1 mbar, 10 min.) in a low-energy plasma cleaning chamber (Pico, Diener electronic GmbH + Co. KG, Germany). The latter treatment was performed in order to remove any organic residual, and to increase the surface energy of the substrate temporarily. A large GeS single crystal (> 3 mm) was placed onto the adhesive tape (Nitto Denko Co., SPV 224P), and was exfoliated multiple times before deposition over the substrate. The last exfoliation step and deposition was done immediately after removing the substrate from the O_2 plasma chamber. The exfoliation was performed with the substrate/GeS/tape stacked heated at 60°C to facilitate the release of flakes onto the substrate.

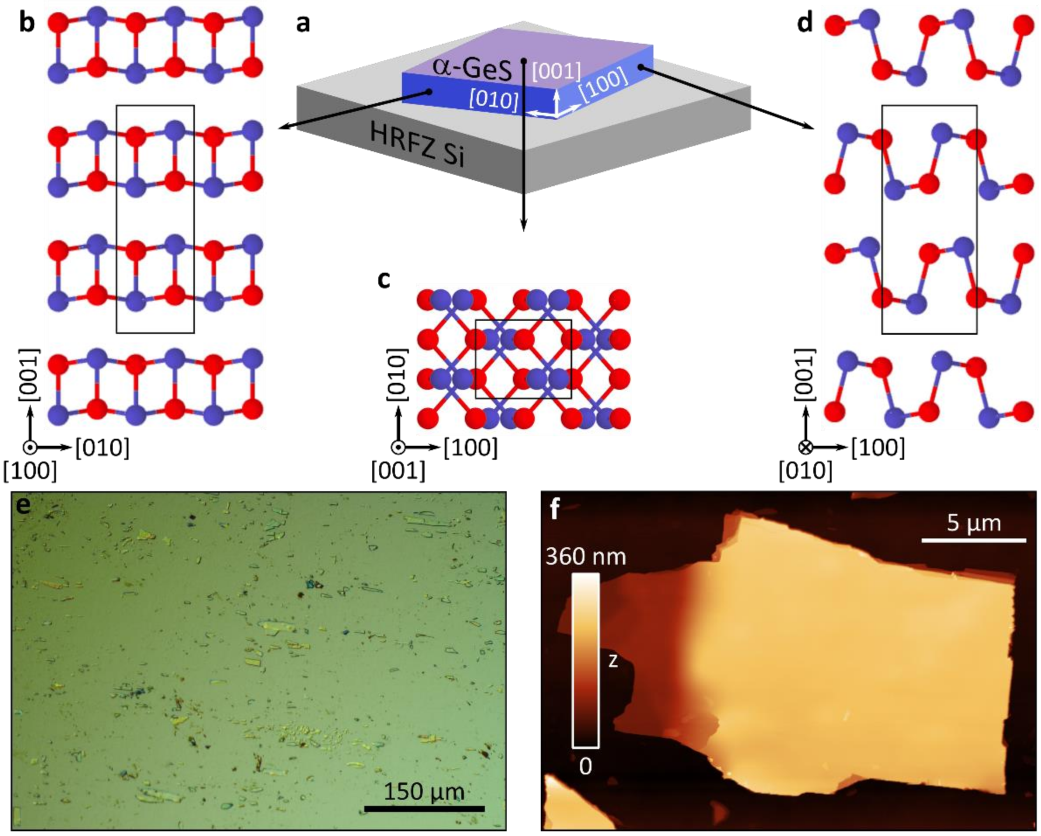

Figure S1. a) Illustration showing a α-GeS flake with indicators of its crystallographic axes. In (b), (c), and (d), the (010), (001), and (100) crystallographic facets of GeS are shown. The crystal structure is generated with the Materials project tool (www.materialsproject.org). e) Optical microscopy image of the sample used throughout this work. f) High-resolution topography image recorded with a NX10 atomic force microscope (Park Systems Corp.).

Note S2: Polarization-resolved Raman spectroscopy

Raman spectroscopy exploits photons that are inelastically scattered by the sample material under investigation. In a nutshell, the frequency difference of the scattered Raman signal as compared to the incident frequency (generated by a pump laser) is referred to as the 'Raman shift' and relates to the landscape of the sample's vibrational modes. Notably, depending on the polarization state of the incident photons relative to the material's crystal axes, the particular excited Raman modes vary in their intensity. Hence, polarization sensitive Raman spectroscopy is well suited for the determination of the crystal axis orientation of a monocrystalline, anisotropic sample such as the GeS flakes studied in this work.

With the geometry inherent to the experiment (that is [001] ∥ z ∥ $k_{in,Raman}$) GeS is anticipated to feature two types of Raman active modes denoted A_g and B_{3g}, which correspond to an in-plane shear vibration of adjacent layers in the zigzag and armchair direction, respectively.[1] Particularly, we employ a co-polarized analyzer in order to measure only the Raman signal with polarization parallel to the (variable) incident polarization. The Raman shift peak intensities may then be described by[1]

$$I(A_g) = (m\cos^2\alpha + n\sin^2\alpha)^2 \tag{S1}$$

$$I(B_{3g}) = k^2(\sin 2\alpha)^2 , \tag{S2}$$

with α the angle between the incident polarization and the [100] crystal direction and *m*, *n* and *k* the proportionality constants derived from individual components of the Raman tensor.

The Raman spectroscopy measurements were performed in backscattering geometry employing a commercial Raman microscope (LabRAM HR Evolution, HORIBA Jobin Yvon GmbH, Germany) together with a 100× objective (Nikon Plan Fluor EPI P; $NA = 0.9$). A Tethered Head H-type module (Innovative Photonic Solutions) serves as the pump laser with up to 100 mW of power and a wavelength of $\lambda = 785$ nm (1.59 eV) specifically selected to be smaller (in terms of energy) than the α-GeS bandgap of $E_{bg} = 1.65$ eV. Furthermore, a CCD detector combined with an 1800 l/mm grating is applied for signal detection, resulting in a spectral resolution of 0.18 cm^{-1}. A linear polarization filter (Glan-Taylor prism) in the detection path ensures the incident and detected light to be always polarized parallel. Finally, the polarization dependent response was probed by rotating the sample by 360° in steps of $\Delta\alpha = 15°$, with the angle α defined as the angle between the incident polarization and a selected edge of the sample (here, parallel to the sample system's x-axis, compare inset of **Figure S2**a).

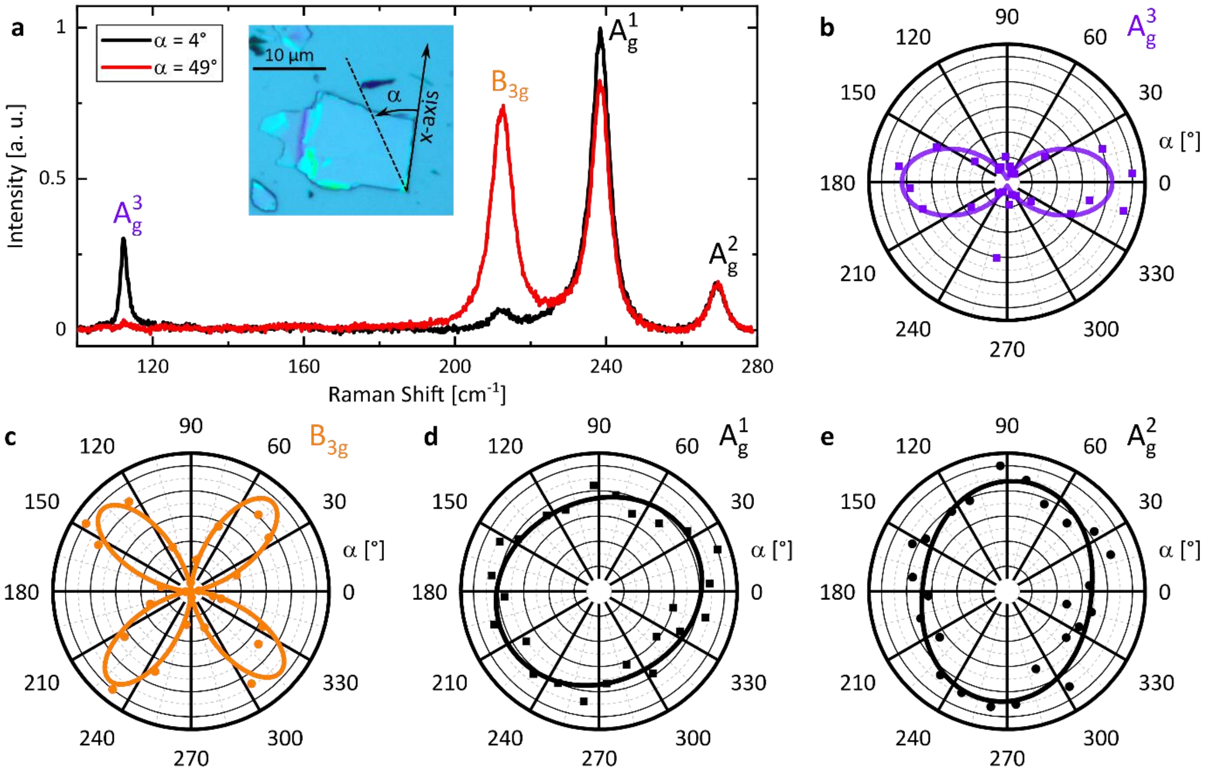

Figure S2. Polarization sensitive Raman response of α-GeS. a) Exemplary Raman spectra taken under incident polarizations of α = 4° and 49°, the angle between the polarization of the exciting field and the GeS crystal x-axis (see inset). The incident wavelength of λ = 785 nm (1.59 eV) was particularly selected to (in terms of energy) fall below the GeS bandgap of 1.65 eV. b,c,d,e) Normalized, polarization-dependent intensities of the Raman modes marked in (a). Particularly the A_g^3 and B_{3g} modes allow for unambiguous assignment of the GeS crystal axes relative to the flake's lateral geometry.

The two exemplary Raman spectra in Figure S2a recorded under the angles α = 4° and α = 49° reveal four Raman peaks at the Raman shifts 112 cm^{-1} (A_g^3), 213 cm^{-1} (B_{3g}), 239 cm^{-1} (A_g^1) and 270 cm^{-1} (A_g^2). Each of the sharp peaks has been successfully assigned to its corresponding Raman mode in literature,[2,3] thus confirming the high quality of the GeS crystal. Moreover, the Raman peak intensities obtained from a series of Lorentz fits to the single spectra have been studied as a function of the incident polarization (radial plots in Figure S2b-e), which are used in the following to determine the alignment of the crystallographic directions of the studied flake.

The A_g^3-mode in Figure S2b features a bowtie shape with the two maxima at around α = 0° and 180°. In particular, a fit of **Equation (S1)** to the experimental data (solid line) confirms the orientation of the maxima of the lobes to be aligned with the flake's x-axis. Hence, we conclude

that for the studied flake, the x-axis is parallel to the [100] crystal direction, since the lobes of the A_g^3-modes are established to be oriented along the [100] direction.[1,2,4,5]

The B_{3g}-mode in Figure S2c exhibits a symmetric clover shape with the four maxima offset by about 45° from the x- and y-axis. The fit applying **Equation (S2)** describes well the experimental data and corroborates the phase offset of $\pi/4$ compared to the A_g^3-mode. The shape and rotation of the B_{3g}-mode (relative to the A_g^3) agree with previous findings in literature.[1]

The A_g^1 and A_g^2-modes in our measurement (Figure S2d,e) do not show distinct maxima but instead exhibit a circular or slightly elliptic shape, which would imply $m \cong n$ for the corresponding Raman tensor elements. In stark contrast, according to Ref.[1] the maxima are expected to hold a bowtie shape (analogous to the A_g^3-mode), in particular with the maximum intensities along armchair ([100]) direction for the A_g^1 and along zigzag ([010]) direction for the A_g^2-mode. We attribute this (reproducible) deviation from literature to the fundamental difference in the experiments that is the excitation wavelength: to the best of our knowledge, previous Raman studies have been conducted with photon energies exceeding the α-GeS direct bandgap of 1.65 eV (for example 2.33 eV (532 nm) by Tan *et al.*,[1] 1.92 eV (647 nm) by Wiley *et al.*,[4] and others[2,5]). Note that we were able to discard other effects on the measured Raman response such as polarization artifacts stemming from our experimental setup or substrate-induced effects. Moreover, repeated measurements conducted on freshly prepared GeS samples feature an identical behavior, thus ruling out degradation effects. Lastly, with our Raman setup we have not been able to properly recreate the measurements with a HeNe laser (λ = 633 nm) without causing substantial damage to the sample, even with incident intensities below 20 µW. In conclusion, the unexpected results for the A_g^1 and A_g^2-modes are subject to further investigation. While the 90° periodic B_{3g} data does not allow for direct differentiation between the crystallographic directions, it does confirm the alignment of one of the in-plane crystal axis

parallel to the GeS flake's x-axis. Finally, the required unambiguous determination of the alignment of the crystal axis is achieved from the A_g^3-mode that, in fact, indicates the [100] crystal direction to be parallel to the x-axis. Moreover, note that the results of the polariton interferometry experiment in itself corroborate the crystal orientation as our experimental findings agree excellently with the theoretical expectations that are based solely on the anisotropic permittivity.

Note S3: GeS THz permittivity

The permittivity of the IV-VI lamellar semiconductor α-GeS in the infrared to THz spectral regime in general is well explored.[3,4,6] It is characterized by a set of optical phonons and can hence be described by a series of Lorentz oscillators. In particular, the vdW material's high anisotropy causes different phonon modes along the three main crystal axes, which renders the permittivity to be expressed by a diagonal tensor with the dissimilar elements $\varepsilon_i(\omega)$ ($i = x, y, z$). The Lorentz model used by Yu *et al.*[6] for the individual components i is a series of three-parameter oscillators

$$\varepsilon_i(\omega) = \varepsilon_{\infty,i} + \sum_k \frac{\omega_{TO,i,k}^2 S_{i,k}}{\omega_{TO,i,k}^2 - \omega^2 - i\omega\omega_{TO,i,k}\gamma_{i,k}}, \qquad \text{(S3)}$$

with the high frequency dielectric constant $\varepsilon_{\infty,i}$ and, moreover, the kth oscillator's strength $S_{k,i}$, transverse optical phonon frequency $\omega_{TO,k,i}$, and corresponding damping $\gamma_{k,i}$. Here, the coordinate system is aligned relative to the crystallographic directions to x ≡ [100], y ≡ [010], and z ≡ [001]. The parameters employed in this work are listed below in **Table S1**. Note that starting from the literature data, we made minor adaptations to few parameters based on our experimental findings. Such changes (summarized in the caption of Table S1) seem reasonable in light of the ultra-high purity and quality of the GeS used in this work (compared to the crystals studied on a macroscopic scale in literature).

Table S1. Oscillator parameters and dielectric constants of GeS. The values used within this work were obtained from literature[6] and slightly adapted to match our experimental data. In particular, the oscillator strength S_1 with E ‖ y was increased from 14 to 17 and the damping γ_1 was decreased by about 40% for E ‖ x and E ‖ y. The weak second oscillator for E ‖ x has an insignificant impact on the permittivity and PhP dispersion and was omitted in the calculations.

	**E ‖ x**	**E ‖ y**	**E ‖ z**
ε_∞	13.6	11.8	12
$\omega_{TO,1}$ **[cm^{-1}]**	257.7	202	237
S_1 **[cm^{-1}]**	7.6	17	7.8
γ_1	0.027	0.022	0.045
$\omega_{TO,2}$ **[cm^{-1}]**	(243)	-	279.7
S_2 **[cm^{-1}]**	(0.4)	-	0.55
γ_2	(0.03)	-	0.04

Note S4: PhP fitting procedure

In the following, the procedure of PhP complex momentum extraction from the polariton interferometry near-field $S_{2\Omega}$ images is laid out. Starting with the experiment, for a fixed excitation frequency optical near-field images of the GeS flake are recorded that (depending on the PhP dispersion) feature characteristic fringes parallel to the flake edges. The spacing between two subsequent maxima/minima for a tip-launched PhP translates to half the PhP wavelength due to the polariton's roundtrip between tip and edge. Owing to the significant high-frequency noise in the FEL-based experiment, typically many (> 50) lines are recorded perpendicular to each of the two relevant edges of the flake, that are later averaged to increase the signal-to-noise ratio. A representative near-field profile extracted perpendicular to the flake edge is presented in **Figure S3**a. The profile shows the common features of a (mostly) tip-launched PhP: Starting from the edge, the near-field signal drops rapidly, followed by the first PhP-related oscillation. Due to edge effects (namely Dyakonov polaritons (PhP edge modes) in type II hyperbolic materials[7]), in the further analysis the first oscillation is always discarded as it holds a smaller wavelength (or a higher momentum) than the rest of the wave pattern. To prepare the experimental data for the fitting, in Figure S3b a high-pass filter is applied (only to

the shaded area in Figure S3a) to remove the DC offset together with potential edge-launched contributions that occur at momenta of half the (main) tip-launched maximum. Note that the HP filtering is a delicate process that could potentially alter the fitting results if applied too invasively, as the frequency component next to the cutoff frequency gets enhanced, effectively. Thus, a rather conservative choice of cutoff frequency is highly recommended and likewise applied in this work.

Continuing with the filtered profile (see Figure S3c), two routes for the fitting open up, each with distinct advantages: Either (I) directly fitting the data in real-space or (II) after additional treatment fitting the data in Fourier space. Below, the two approaches are described and compared, as both have been used throughout this work.

(I) Naturally, the filtered experimental data may be fitted using the common analytical description of an edge-reflected polaritonic wave[8]

$$f(x) = A \frac{1}{\sqrt{x}} \exp\left[-2\,\mathrm{Im}(k)x\right] \sin[2\,\mathrm{Re}(k)(x - x_0)]\,, \qquad \text{(S4)}$$

with the fitting parameters being the amplitude A, coordinate, x_0, accounting for the phase, and complex momentum, k. The formula (S4) describes a damped sine wave also accounting for the PhP geometrical spreading (red curve in Figure S3c). From the fitting procedure, the complex momentum is obtained directly. As a requirement for the fitting to produce feasible results, the profile has to cover several PhP oscillations. A similar fitting shape was employed to the profiles at the selected frequencies $\upsilon_{1\text{-}3}$ for both, the experimental and simulated data.

(II) For the second approach, fitting in Fourier space, the correction for the PhP spreading across the flake surface has to be applied to the data, as it is not incorporated in the Lorentz fit function (compare $1/\sqrt{x}$-factor in **Equation (S4)**). Thus, the $S_{2\Omega}$ profile is stretched by $\sqrt{x}$ and the resulting $S_{2\Omega}$* distribution transformed into Fourier space using a Hanning window function. The result is displayed in Figure S3d. Note that the x-axis was rescaled from spatial frequency

to tip-launched polariton momentum k* (by a factor of π, effectively). Finally, the Fourier transformed data is fitted using a Lorentz function with the center frequency corresponding to the PhP *Re*(*k*) and the half width at half maximum equal to *Im*(*k*).[9] The FFT fitting technique appears to provide more reasonable results in case of strong PhP damping (*i.e.*, only few field oscillations visible within the profile). For that reason, this technique was widely employed in this work, namely the dispersion in Figure 3a as well as the figure of merit, life time, and light confinement in Figure 4).

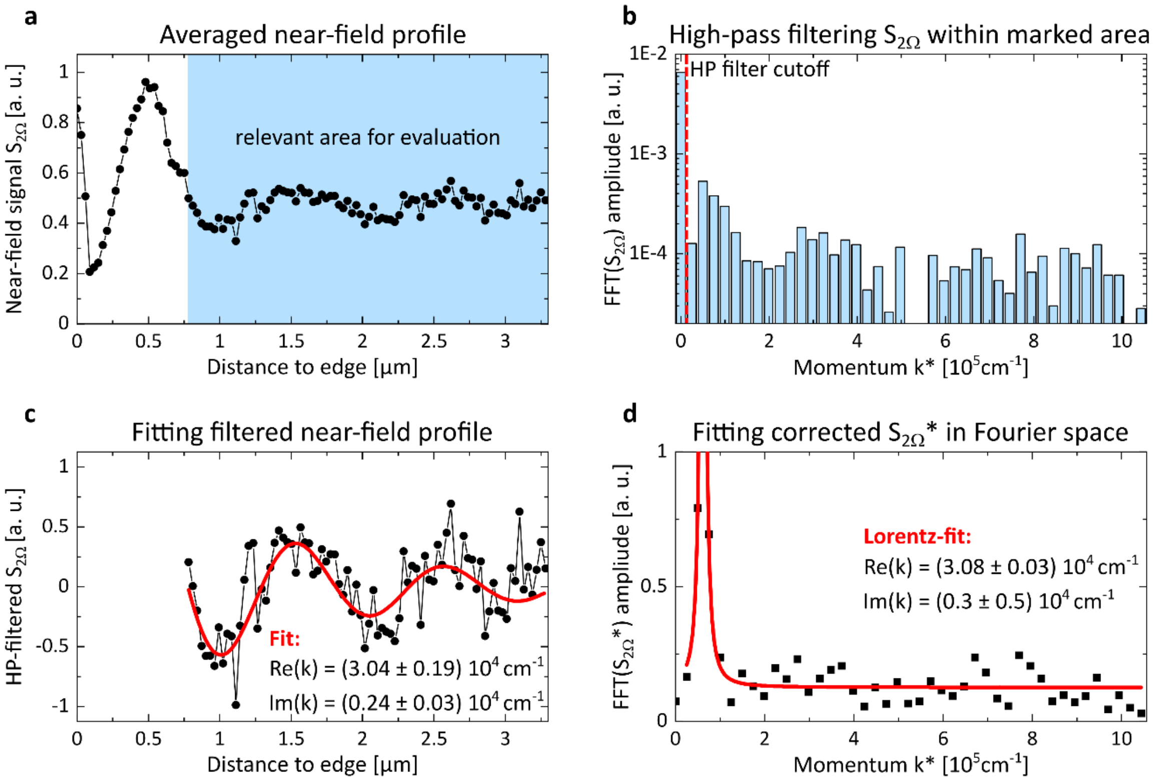

Figure S3. Extraction of the PhP complex momentum from near-field profiles. a) Averaged near-field $S_{2\Omega}$ profile perpendicular to a GeS flake edge extracted from a 2D NF image. The white area is omitted in the following. b) Fourier-transformed representation of the NF profile (blue area in (a)) as used for high-pass filtering. The red dashed line relates to the HP filter cutoff frequency, here removing the DC component from the profile. c) Filtered NF profile fitted in real space using Equation (S4) (red curve). d) Fourier-transform of the treated $S_{2\Omega}$* profile. The red curve presents a Lorentz fit to the data through which the PhPs *Re*(*k*) and *Im*(*k*) are obtained.

Note S5: Noise analysis in FEL-based polariton interferometry

As evident from the near-field images and profiles in Figure 2, the applied polariton interferometry setup suffers from a high noise level, mostly introduced by the free-electron laser. For that reason, measures are taken to enhance the signal-to-noise ratio (SNR) in the data analysis process and to extract the PhP field oscillations against the background noise. In the following, the noise levels of the data together with fitting errors during individual steps of the data analysis are detailed, as illustrated in **Figure S4**.

To that end, the untreated near-field $S_{2\Omega}$ image of the 224 nm-thick GeS flake recorded at ν = 7.33 THz in Figure S4a presents the starting point of the data processing: On the one hand, the image exhibits a high-frequency noise due to the FEL's pulse intensity jitter, and on the other hand, a slow fluctuation of the incident power is evident, induced likely by thermal drifts of the resonator. Thus, two profiles were extracted at the marked positions each along [010] direction with a width of 32 px, that will be analyzed individually. The noise of the near-field signal $S_{2\Omega}$ is investigated at the red-shaded locations that correspond to the GeS and Si dielectric responses (away from PhP wave) at the positions of the profiles. The obtained values for the mean signal, the RMS noise, and SNR are summarized in the table in Figure S4c.

Next, the extracted, averaged near-field $S_{2\Omega}$ profiles are displayed in Figure S4b, with the profile 1 (red) exhibiting a lower overall signal strength than the profile 2 (black). Again the SNR is evaluated on the Si and GeS within the red-shaded areas and the values depicted in the table in Figure S4c. Here, we find a substantial increase of the SNR by a factor of 10 due to the averaging process reducing the random noise. To access the SNR concerning the PhP field oscillations on top of GeS dielectric response, a Fourier transformation of the $S_{2\Omega}$ profile in the indicated area of Figure S4b is performed, with the FT amplitude as a function of spatial frequency displayed in Figure S4d: In fact, the transformed profiles have a distinct peak at the

frequency corresponding to a PhP with the momentum anticipated by the analytical model. Comparing the peak amplitudes (marked by the dotted lines) and the mean value of the noise floor (solid and dashed horizontal lines) gives a PhP-related SNR^{PhP} of 4.7 (4.9) for profile 1 (profile 2). Also note the low-frequency cutoff indicated by the hatching that would have been typically applied for this data set in case of a momentum-space fitting.

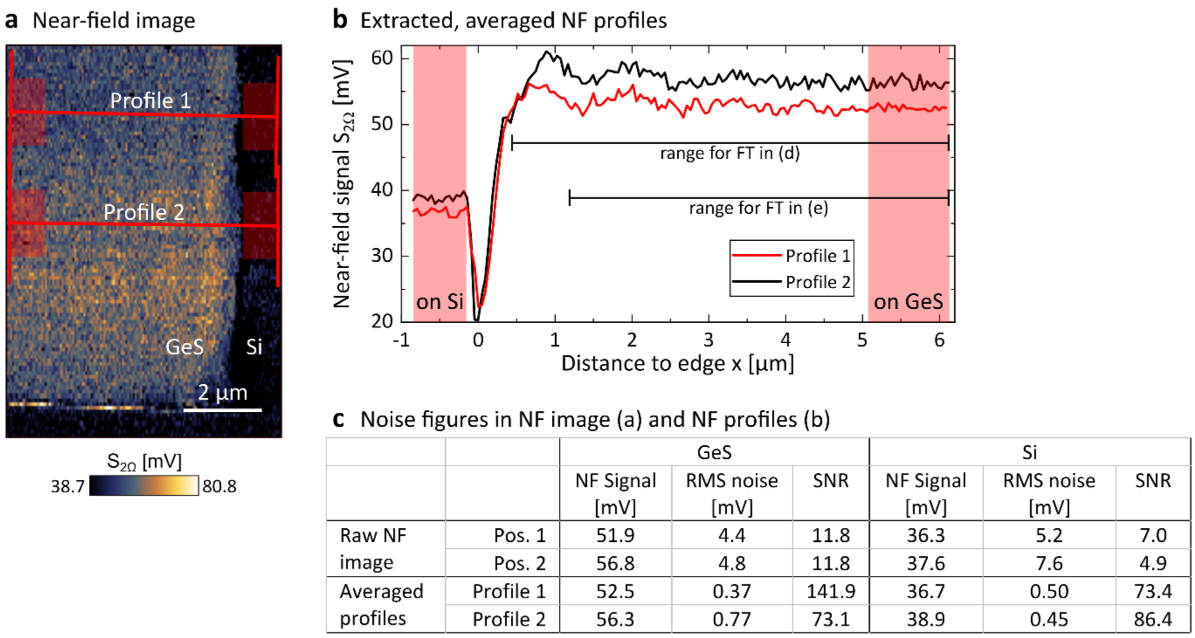

c Noise figures in NF image (a) and NF profiles (b)

		GeS			Si		
		NF Signal [mV]	RMS noise [mV]	SNR	NF Signal [mV]	RMS noise [mV]	SNR
Raw NF image	Pos. 1	51.9	4.4	11.8	36.3	5.2	7.0
	Pos. 2	56.8	4.8	11.8	37.6	7.6	4.9
Averaged profiles	Profile 1	52.5	0.37	141.9	36.7	0.50	73.4
	Profile 2	56.3	0.77	73.1	38.9	0.45	86.4

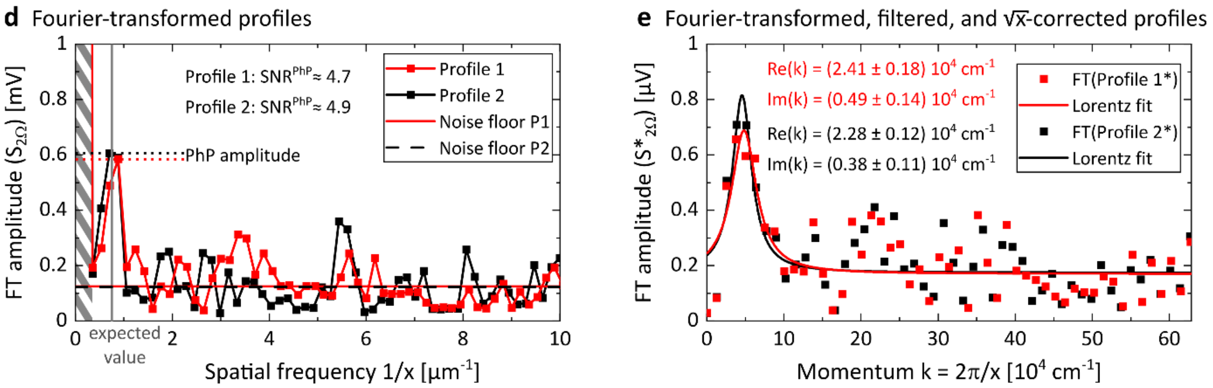

Figure S4. Examination of the noise in the FEL-based polariton interferometry data analysis. a) Untreated near-field $S_{2\Omega}$ image recorded at an excitation frequency of ν = 7.33 THz. The lines present the positions and averaging width of two extracted near-field profiles. The red-shaded areas on Si and GeS have been chosen for the evaluation of the different SNR values of the raw data. b) Near-field profiles extracted from (a), averaged over 32 px, each. The marked areas again represent where SNR values of the dielectric response of Si and GeS have been obtained. c) Table summarizing the signals, noise level, and SNR of the raw image in (a) and the averaged profiles in (b), both for Si and GeS. d) Fourier transform amplitude of the two NF profiles in (b) in the indicated range (complete GeS surface). The noise floor of each profile (solid red line and dashed black line) was determined by averaging and the PhP peak amplitude (dotted lines) read from the data. The grey vertical line indicates the expected tip-launched

PhP's spatial frequency and the hatched area practical high-pass filter cutoff frequency. e) Fourier-transformed near-field signal amplitude after high-pass filtering and correction for the geometrical spreading, after discarding the first peak [in real-space, see (b)]. The PhP momentum was obtained through fitting a Lorentzian to the data (described in previous section S4).

Regarding the eventual PhP momentum extraction, as described earlier in Note S4, we (i) chose a different range for the FT (discarding the first peak or fringe), remove the very low-frequency components through a high-pass filter, and (iii) correct the 1D profile for the geometric spreading in 2D by applying a factor of $\sqrt{x}$ to the data. The FT amplitude of the resulting $S^*_{2\Omega}$ profile is presented in Figure S4e as a function of momentum k. The complex momentum is then obtained by fitting Lorentzians to the data. The final error margin of the momentum is then given by the 99 % confidence interval of the fitting, with typical values in the range of $\Delta Re(k)/Re(k) < 10$ % and $\Delta Im(k)/Im(k) = 25$ %.

Note S6: Origin of anomalous dispersion

The dispersion of PhPs found in a GeS slab shows several regions of anomalous behavior in combination with negative group velocity. As discussed in the main manuscript, similar effects have been observed for coupling of polaritons to extrinsic resonances,[10] however, no such external vibrational states are present in our system. In contrast, we find the novel spectral overlap between the two intrinsic z-phonons with the in-plane reststrahlen bands to induce the dispersion back bending. To investigate the underlying mechanism, in the following the interplay between the air, silicon, and GeS permittivities and their impact on the PhP dispersion are investigated theoretically. To that end, we study the individual contributions to the PhP dispersion stemming from the two interfaces, air/GeS and GeS/Si, respectively.

The PhP momentum inside the GeS slab may be derived from the Fabry-Pérot condition for an electromagnetic wave experiencing multiple reflections inside the slab, so that it linerly depends on the sum of the phases of the Fresnel reflection coefficients of the interfaces.[11] Therefore, the two frequency-dependent terms from Equation (1) that are directly proportional to the phase of

the Fresnel coefficients are examined for the incident polarizations parallel to the GeS [100] and [010] crystal directions that write:

Along [100] direction: $$i\sqrt{\frac{\varepsilon_z}{\varepsilon_x}}\arctan\left(\frac{i\,\varepsilon_j\sqrt{\frac{\varepsilon_z}{\varepsilon_x}}}{\varepsilon_z}\right), j = air, Si\ , \qquad \text{(S5)}$$

Along [010] direction: $$i\sqrt{\frac{\varepsilon_z}{\varepsilon_y}}\arctan\left(\frac{i\,\varepsilon_j\sqrt{\frac{\varepsilon_z}{\varepsilon_y}}}{\varepsilon_z}\right), j = air, Si\ , \qquad \text{(S6)}$$

with ε_x, ε_y, and ε_z the GeS permittivity diagonal tensor elements and ε_j (j = *air*, *Si*) the permittivity of the isotropic air superstrate and silicon substrate.

The curves calculated from the **Equation (S5)** and **(S6)** above are displayed in **Figure S5** with the colors reflecting the incident polarization and the solid/dashed lines the respective interface. The values obtained for the air/GeS interface (contributing to the real part of the PhP momentum) for the two incident polarizations show similar behavior (dashed curves): both exhibit a negative sign outside of their respective GeS in-plane reststrahlen bands. With increasing frequency ν, the first zero-crossing occurs at the TO phonon frequencies. From there, the values increase monotonically until they smoothly drop down and change sign around the LO phonon frequencies. In fact, this shape closely resembles the PhP dispersion for anisotropic polar materials, where the RBs do not overlap (for instance compare α-MoO_3, α-V_2O_5, and hBN).

In contrast, the contribution to the PhP momentum related to the reflection at the GeS/Si interface (solid curves in Figure S5) is far from trivial. Particularly note that the difference between the curves of similar color solely stems from the replacement of $\varepsilon_{air} \approx 1$ with $\varepsilon_{Si} \approx 11.68 + 0.0001i$, which effectively increases the weight of the $\sqrt{\varepsilon_z/\varepsilon_i}/\varepsilon_z$-term ($i$ = x, y) within the

tan^{-1}-function. The latter term is particularly interesting due to its intricate non-linear behavior caused by the optical phonons in [001] direction in GeS.

We conclude that the interplay between the ε_z and ε_x (and similarly ε_y) GeS permittivity tensor elements induces the non-monotonous behavior of the PhP momentum: the frequency-dependent reflection of the electromagnetic wave at the GeS/Si interface (that is constituted by the different GeS and Si permittivity elements) largely varies in the studied frequency regime and hence alters the PhP momentum dictated by the Fabry-Pérot resonance condition.

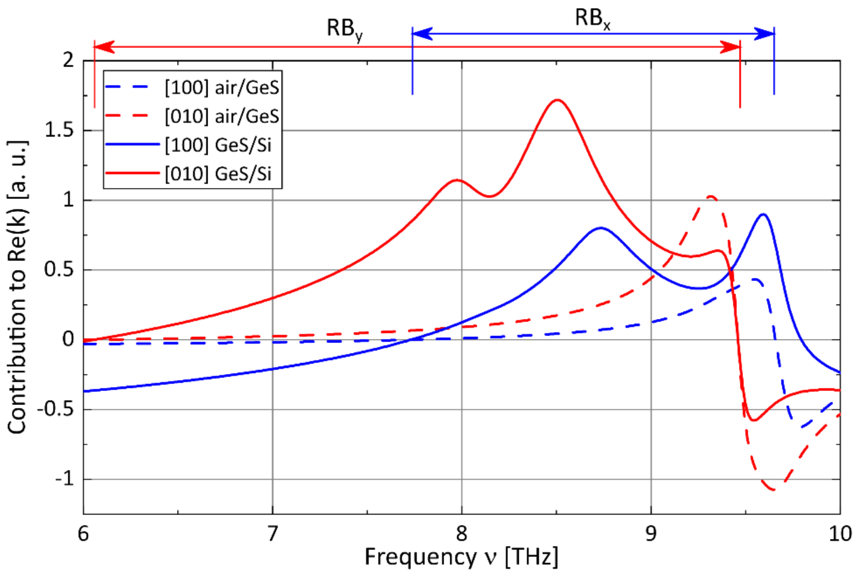

Figure S5. Contributions of the separate em-wave reflections at the air/GeS and GeS/Si interfaces to the PhP momentum. The curves calculated from Equation (S4) and (S5) are proportional to the phase of the Fresnel coefficients, linearly contributing to the PhP momentum. The solid (dashed) lines represent the GeS/Si (air/GeS) interface, with the polarization of the incident light parallel to the GeS [100] ([010]) direction for the blue (red) curve, respectively. Moreover, spectral locations of the in-plane reststrahlen bands RB_x and RB_y are indicated.

Next, in order to illustrate the impact of ε_z on the in-plane PhP momentum, we calculate for several compositions of $\varepsilon_z(\nu)$ the polariton's $Re(k)$ in **Figure S6**. More specifically, we compare the PhP momenta of a 100 nm-thick slab of GeS for the three cases of the complex-valued $\varepsilon_z(\nu)$ displayed in Figure S6a: The solid line representing the original permittivity ε_z with the two optical phonons, the dashed line only considering the strong z-phonon at 7.1 THz, and lastly, the dotted curve with $\varepsilon_z(\nu) = \varepsilon_{z,\infty}$ featuring no phonons. The impact on the PhP dispersion in

[010] direction is portrayed in Figure S6b: Using the original permittivity ε_z, a dispersion back bending is found in two distinct spectral areas, around 8.1 THz and 8.7 THz (identical to the dispersion in the main manuscript). Discarding the weak z-phonon at 8.4 THz (dashed line), the area of anomalous dispersion around 8.1 THz vanishes, while the second one around 8.7 THz becomes larger, effectively. From this observation we conclude, that the back-bending effect at the lower frequency of 8.1 THz is essentially induced by the weak z-phonon at 8.4 THz. Moreover, by further comparison to the dispersion curve with constant positive ε_z, we find the strong z-phonon at 7.1 THz to be responsible for the larger area of anomalous dispersion in the original dispersion curve.

A similar analysis for the PhP dispersion in [100] direction is presented in Figure S6c, where a comparable response of the dispersion can be observed, except for the impact of the weak z-phonon being negligible when it comes to the back bending. By removing the weak z-phonon from the permittivity ε_z (dashed curve), the width of the area of anomalous dispersion increases, while without z-phonons (dotted curve) the anomalous PhP dispersion disappears. The difference in the number of spectral areas with anomalous dispersion in [100] vs. [010] direction can be attributed to the difference in ε_x and ε_y: Inside the respective reststrahlen bands, throughout we have $\varepsilon_x \ll \varepsilon_y$ and $\varepsilon_x \ll \varepsilon_z$, which simplifies the $\sqrt{\varepsilon_z/\varepsilon_x}/\varepsilon_z$-term as compared to the $\sqrt{\varepsilon_z/\varepsilon_y}/\varepsilon_z$-term.

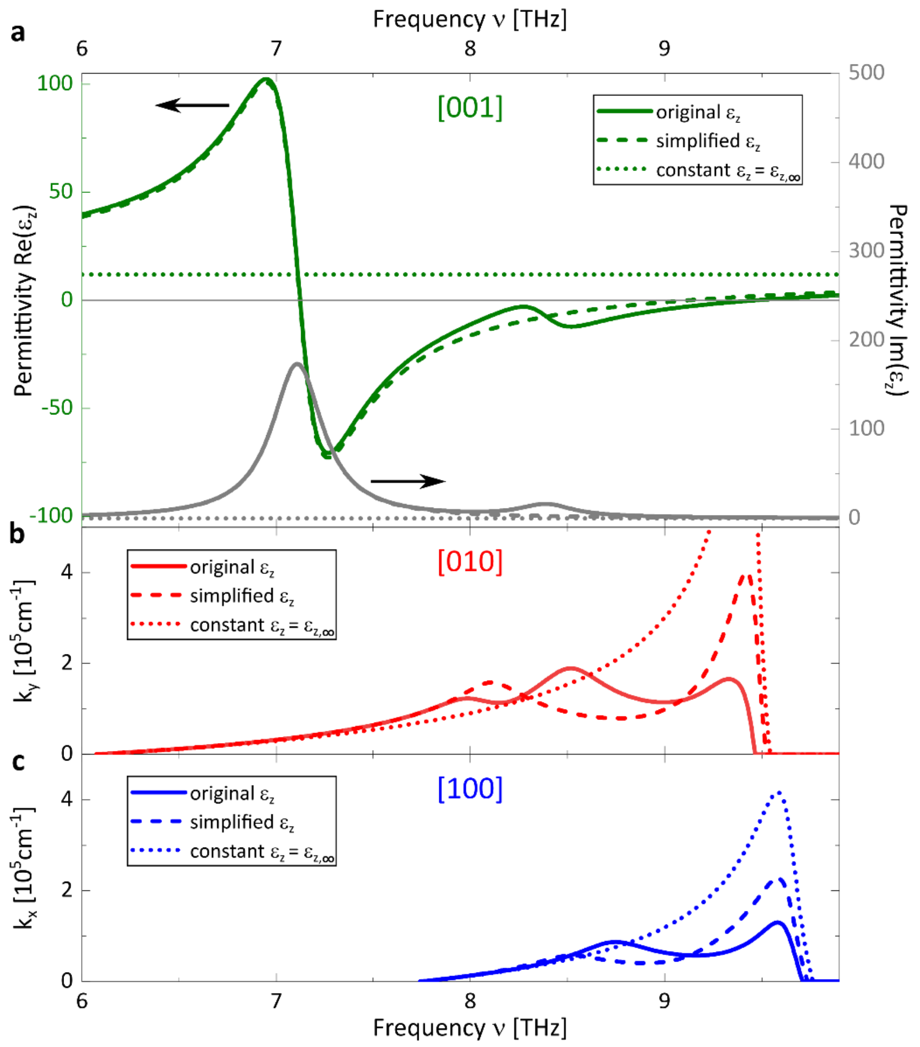

Figure S6. Influence of the optical phonons in [001] direction on the in-plane PhP dispersion. a) Real (olive) and imaginary part (grey) of the natural and modified GeS permittivity in [001] direction. The solid curves represent the permittivity from literature with the two optical phonons. The dashed curves correspond to only the strong z-phonon, while the dotted curves relates to a constant dielectric function. b) Analytically calculated dispersion in [010] direction using the permittivity ε_z displayed in (a). c) Same as in (b) but for the [100] direction.

Lastly, the impact of the substrate's permittivity on the PhP dispersion is considered. As seen in Figure S5, the phase matching conditions at the interfaces between GeS slab and the substrate and superstrate have a large impact on the layout of the PhP dispersion in the slab (compare the dashed and solid curves). Notably, the permittivity of the substrate appears in the analytical expression for the PhP dispersion in form of a factor in the argument of the $\tan^{-1}$-function, as was mentioned earlier. Through selection of different values for ε_3 and subsequent calculation of the resulting PhP dispersion, we visualize the non-trivial effect in **Figure S7**. The

permittivities of the GeS are repeated in Figure S7a, alongside four different, constant permittivities for the substrate, ε_3 = 1, 5, 11.68, and 25 (solid lines with black–yellow color gradient). The resulting PhP dispersion along [010] direction is presented in Figure S7b for a 100 nm-thick GeS slab with the color gradient following the one in (a). Firstly, as anticipated from the dashed lines in Figure S5, the dispersion of PhP in a suspended GeS flake (air/GeS/air, black curve) does not feature any back bending. With increasing ε_3 the characteristic cut in the PhP dispersion (previously assigned to the strong z-phonon) appears. The cut shifts towards lower frequencies and increases in spectral width. For $\varepsilon_3 > 5$, the second back bending area occurs at around ν = 8 THz, showing a similar behavior with increasing ε_3.

Regarding the PhP dispersion along [100] direction in Figure S7c, similarly to the [010] direction no anomalous dispersion is found for ε_3 = 1. With an increasing permittivity ε_3, a single back bending area emerges, also shifting towards lower frequencies and increasing in width. Intriguingly, a high permittivity of the substrate introduces a minor bump near ν = 8.1 THz to the dispersion, which appears to be a precursor to a back-bending effect. As observed in the manuscript in Figure 3a, this is not evident from the PhP dispersion with silicon as a substrate, however is in line with the findings along [010] direction in (b).

Overall, the effect of the substrate's dependence on the in-plane PhP dispersion are quite exotic compared to other PhP-hosting materials, which we attribute to the z-phonons of GeS: Typically the shape of the dispersion remains constant and only the absolute values vary. However in case of GeS, we find the dispersion back bending to be induced by the z-phonons and mediated by the substrate, the latter thus being a parameter for control of the position and width of the dispersion back bending areas.

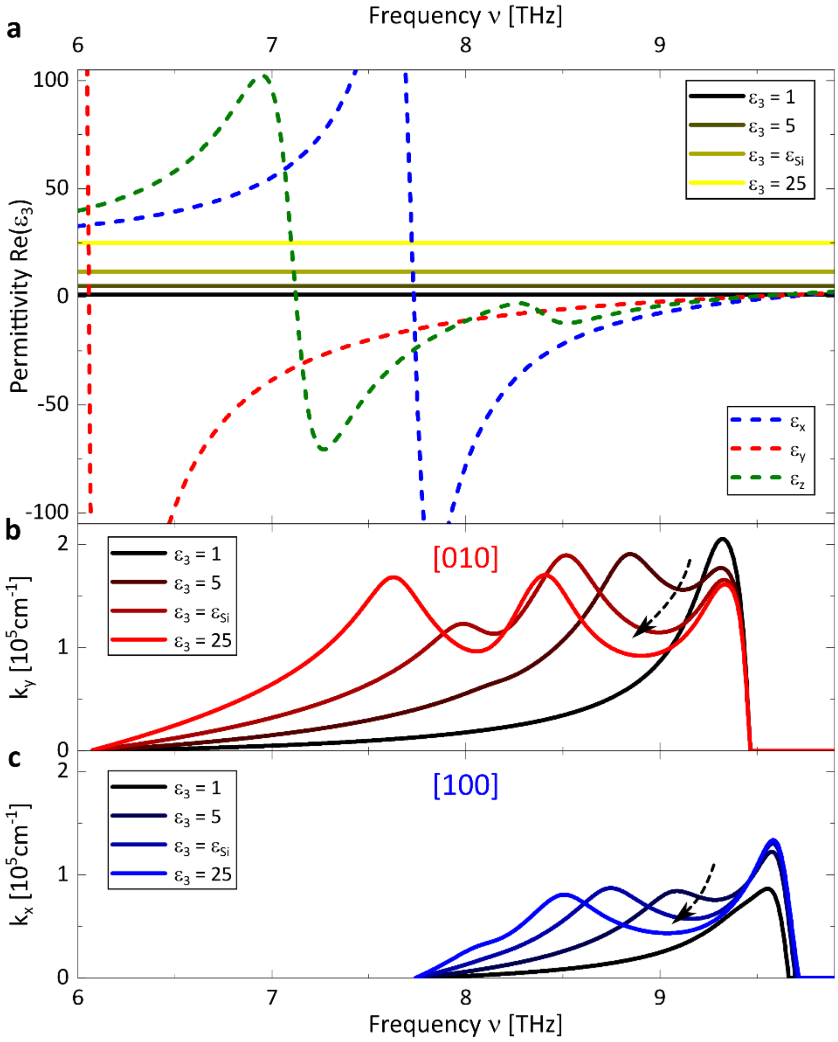

Figure S7. Influence of the substrate's permittivity ε_3 on the GeS in-plane PhP dispersion. a) Overview of the different permittivities contributing to the PhP dispersion in GeS (apart from ε_1 = 1). The dashed lines correspond to the diagonal tensor elements of the GeS permittivity and the solid lines following the black–yellow color gradient denote different values of the substrate's isotropic permittivity ε_3. b,c) PhP dispersion calculated for a 100 nm-thick GeS flake on different substrates along [010] and [100] direction, respectively. The dashed arrows are guides to the eye regarding the progression of different back bending areas with increasing substrate permittivity.

Note S7: 3D representation of the PhP field distribution

Motivated by the multiphonon effects in GeS permittivity and the resulting PhP dispersion curve, in the following we investigate the PhP electric field spatial distribution within the layered system. To that end, we perform full-wave electromagnetic simulations and analyze the out-of-plane electric field component $Re(E_z)$ (see Experimental Section in the main manuscript

and compare with Duan *et al.*[12]). In particular, we show the electric field of a section within the (x, y)-plane at the position z = 234 nm (*i.e.*, 10 nm above the GeS surface, see **Figure S8**) together with cross sections through the (x, z) and (y, z)-plane (assuming the same coordinate system as in the main manuscript). Note that the driving dipolar source introduces "far-field" components which interfere with the PhP fields and produce phase shifts (or field discontinuity) mostly at the air/GeS interface.

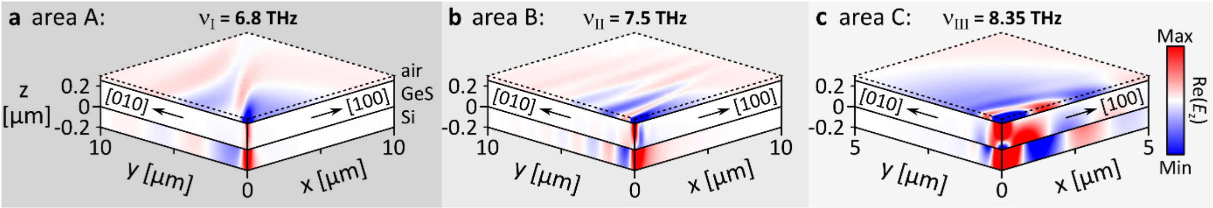

Figure S8. Three-dimensional depiction of the simulated PhP electric field component $Re(E_z)$ in a GeS slab for different frequencies ν in the spectral areas A-C. The layered system consists of a 225 nm-thick GeS slab in between a semi-infinite HRFZ Si substrate and air superstrate with the driving dipole positioned 265 nm above the GeS surface. The top (x,y-) facet presents the field 10 nm above the air/GeS interface (z = 234 nm) and the coordinate system is aligned to the GeS crystallographic directions so that x ≡ [100], y ≡ [010], and z ≡ [001].

The diagram in Figure S8a depicts the simulated $Re(E_z)$ within the GeS slab at an excitation frequency of ν_I = 6.8 THz in area A. The field in (x, y)-plane reveals a PhP wave pattern: Starting from the (0, 0)-position below the dipole, the PhP propagates with hyperbolic wave fronts along [010] direction. The long-wavelength oscillation along [100] direction presents the dipole's "far-field" background (compare simulation in Figure 3b at ν_A = 7.02 THz). This background becomes evident when considering the (x, z)-plane, where the confined field inside the GeS and Si only exists at the origin and decays rapidly along x. This strong decay of the $Re(E_z)$ at the origin relates to the finite width of the PhP propagating mainly along y-direction, revealing the quick field decay in GeS and especially Si perpendicular to the main direction of propagation. Lastly, the (y, z)-cross section features the PhP field projection along the main propagation direction: While the chosen color scale hides the field inside the GeS slab, the field inside the Si substrate shows a damped oscillation along y-direction exponentially decaying along -z-direction. The mismatch between this wave pattern as compared to the field in air is

solely caused by the dipole's background contribution. A more detailed investigation of the field inside the GeS slab will be laid out in the subsequent section S8, where we find the fields inside GeS and Si at the interface to be in phase.

The in-plane field in Figure S8b simulated at ν_{II} = 7.5 THz in area B similarly reveals PhP with hyperbolic wavefront sector centered along [010] direction, superposed by the background field. Notably, the wavelength is shorter and the lateral spread is higher as compared to the PhP field distribution at ν_{I} = 6.8 THz displayed in Figure S8a. In the (x, z)-plane inside the GeS, a highly damped mode with large momentum occurs, that we will later (section S8) attribute to the l = 1 PhP mode that is expected to propagate along [100] direction. On the other hand, in the (y, z)-cross section we again find the oscillations revealing the in-plane hyperbolic PhP propagating along [010] direction. Note that here, the field inside the GeS and the Si are both visible and out of phase by π. This change in comparison to area A can be attributed to the GeS permittivity component $Re(\varepsilon_z)$ becoming negative.

Finally, the diagram in Figure S8c reveals the $Re(E_z)$ for a frequency of the driving dipole of ν_{III} = 8.35 THz (area C). The (x, y)-plane presents a field distribution of a PhP propagating with elliptical wavefronts with a longer wavelength in x compared to y direction. Note that the canalization effect found at ν_C = 8.57 THz is not apparent under the present permittivity $\varepsilon(\nu)$. In the (x, z)-cross section a propagating wave is found along [100] direction in both GeS and Si, however with an strong phase offset. The same effect occurs along [010] direction in the (y, z)-plane, with the PhP holding a significantly shorter wavelength and decaying faster with lateral distance from (x=0, y=0).

In summary, we visualized the influence of the different permittivity tensor components within the three distinct areas A-C on the PhP properties in 3D. In particular, the in-plane field distribution in all three areas matches our findings obtained from the experiment, which is

sensitive only to the PhP field above the GeS surface. Below, the simulated fields in areas A and B are analyzed regarding the l = 1 higher order PhP mode.

Note S8: 90° Rotation of the l = 1 mode at ν = 7.1 THz

The difference between PhPs belonging to the spectral areas A and B (as denoted in the main manuscript, Figure 1 and 3) originates from the change of sign of $Re(\varepsilon_z)$ due to the optical phonon mode at 7.1 THz along the [001] direction. From the analytical model, we expect the PhP mode with quantization index l = 0 to be essentially unaffected by the change of sign, whereas the orientation of the IFCs of the mode with l = 1 in areas A and B is mutually rotated by 90°. While the orientation of the IFCs of the mode with l = 0 is confirmed by both our experiment and the full-wave numerical simulations, experimental evidence of the rotation of the l = 1 mode is lacking. Hence, the latter is addressed in detail in the following by revisiting the simulated field distribution cross-sections at 6.8 and 7.5 THz (from Figure S8a,b) and through calculation of the IFC specifically around the TO phonon frequency $\nu_{TO,[001],1}$ (*i.e.*, the zero crossing of $Re[\varepsilon_z]$).

In order to investigate the orientation of the IFC of the high momentum mode with l = 1 in the spectral areas A and B, we extract line profiles from the data arrays of the simulated out-of-plane electric field $Re[E_z(x,z)]$ and $Re[E_z(y,z)]$ distributions at the frequencies ν = 6.8 and 7.5 THz, respectively. The line profiles are taken in the center of the 224 nm-thick GeS slab along both the [100] and [010] in-plane direction (see **Figure S9**a,b) in order to better visualize the higher order PhP modes, which mostly reside inside the bulk. Figure S9c shows the line profiles in the interval from 0 to 4 μm at ν_I = 6.8 THz, represented by the black and orange curves along [100] and [010] directions, respectively. Here, the field along [100] direction is solely constituted by the rapidly decaying peak at the origin (no field oscillations visible) that relates

to the finite width of the PhP propagating mainly along [010]. This observation is in line with the IFCs in Figure 3b, that indicates PhP propagation along [100] to be forbidden. On the other hand, in [100] direction we find a superposition of oscillations with two different wavelengths, of which the high momentum oscillation is of particular interest: From the profile, we identify the wavelength of the rapidly decaying wave to be about $\lambda_{[010]}^{l=1} \approx 0.31\ \mu m$, corresponding to a momentum of $k_{[010]}^{l=1} \approx 2.0 \times 10^5\ cm^{-1}$. This finding is matching well the calculated value from the Equation (1) for the *l* = 1 mode yielding the momentum $k_y^{l=1} = 1.92 \times 10^5\ cm^{-1}$. In conclusion, the simulated out-of-plane field distributions inside the GeS slab are consistent with the IFCs given by Equation (1), particularly with respect to the values of momentum of the *l* = 0,1 PhP modes and their propagation direction.

At ν_{II} = 7.5 THz, the lateral profiles inside the GeS slap presented in Figure S9d both show electric field $Re(E_z)$ oscillations that may be attributed to PhP modes: In [100] direction, a rapidly decaying mode with wavelength $\lambda_{[100]}^{l=1} \approx 0.81\ \mu m$ ($k_{[100]}^{l=1} \approx 0.78 \times 10^5\ cm^{-1}$) is found that can be assigned to the *l* = 1 mode calculated to be $k_x^{l=1} = 0.815 \times 10^5\ cm^{-1}$. Note, that no second PhP mode is featured within the profile in [100] direction. On the other hand in [010] direction (orange curve in Figure S9d), we observe a field oscillation with an approximate wavelength of $\lambda_{[010]}^{l=0} \approx 2.06\ \mu m$ and momentum $k_{[010]}^{l=0} \approx 0.30 \times 10^5\ cm^{-1}$. Again, this momentum matches very well the anticipated analytical value of $k_y^{l=0} = 0.291 \times 10^5\ cm^{-1}$, and is thereby confirmed to correspond to the *l* = 0 PhP mode. In summary, at 7.5 THz we find the simulated field distributions to agree with the analytical expectations of the propagating hyperbolic *l* = 0 and *l* = 1 PhP modes that are propagating along orthogonal in-plane directions.

Note that, as the polariton's momentum $k_{\parallel}$ is conserved at the interfaces and throughout the system, similar field oscillations corresponding the *l* = 1 modes are present inside both the air and the silicon. Due to the significant influence of the far-field contribution of the exciting

dipole together with the modes' highly evanescent nature, there such higher order PhPs are not as easily observed, hence we chose to investigate these modes inside the GeS, where the impact of the exciting dipole field is significantly smaller.

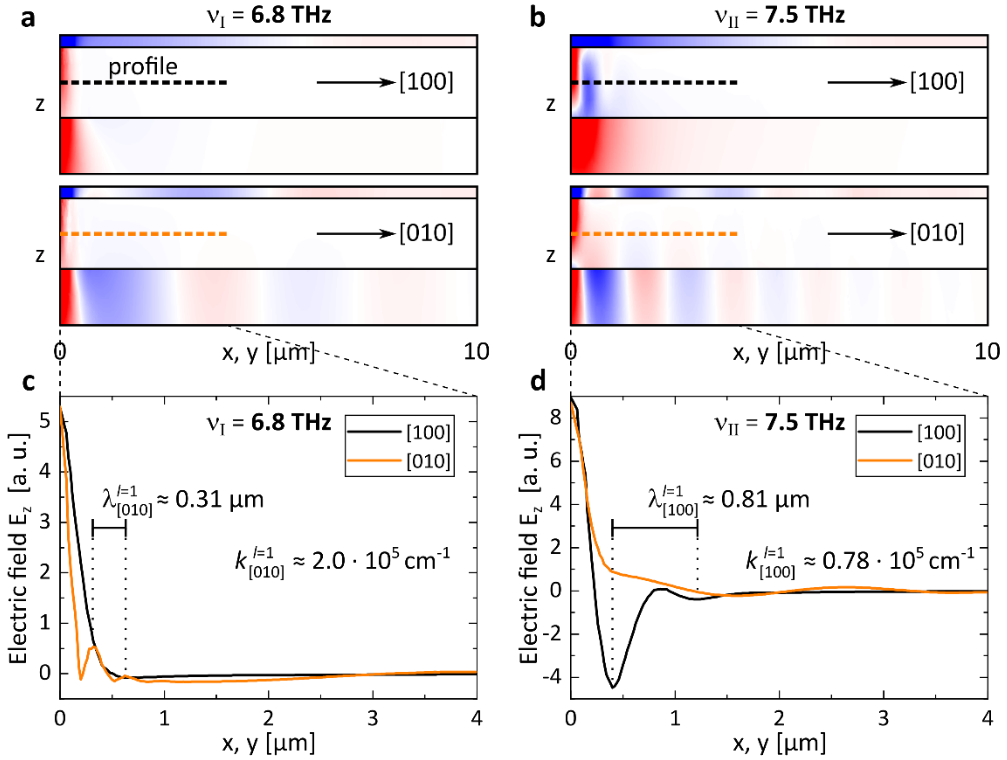

Figure S9. In-depth investigation of the PhP modes inside the GeS slab. a) (x, z) and (y, z)-cross-sections of the full-wave simulated field distribution E_z of the air/GeS/Si system with an exciting frequency of $\nu_I = 6.8$ THz (reproduced from Figure 3). Lateral profiles of 4 µm length have been extracted at the center of the GeS slab at the marked positions along both in-plane directions. b) Same as in (a), except with a different excitation frequency of $\nu_{II} = 7.5$ THz. c) E_z profiles extracted from (a). The profile in [010] direction (orange) features a superposition of two decaying waves of different wavelength, of which the smaller may be attributed to the $l = 1$ PhP mode (the long wavelength corresponds to the $l = 0$). d) Analogous to (c), but with the profiles extracted from (b) at 7.5 THz excitation frequency. Both profiles show an exponentially decaying wave, with the field oscillation in [100] direction related to the $l = 1$ PhP mode that is propagating perpendicular to the $l = 0$ mode.

Next, the frequency-dependent rotation of the IFCs around the zero-crossing of the permittivity Re(ε_z) in z-direction is investigated theoretically. To that end, we calculate the IFCs in the frequency range of 7.105 – 7.141 THz (237 – 238.2 cm^{-1}) specifically for $l = 1$ and propagating solutions $Re(k) > Im(k)$ only. Note that the frequency where the zero crossing in $Re(\varepsilon_z)$ appears is slightly blue-shifted from the TO phonon frequency $\nu_{TO,[001],1} \approx 7.105$ THz (237 cm^{-1}) towards 7.118 THz (237.432 cm^{-1}), due to the damping term in the three-parameter Lorentz oscillator

model. The calculated IFCs in the frequency range stated above are presented in **Figure S10**, where the anticipated rotation of the hyperbola's major axis is found. More precisely, starting in area A with increasing frequency the hyperbolas become more narrow (meaning the possible k_x-components decrease with frequency) up until 7.114 THz (237.3 cm^{-1}), where the momentum $k_y = 2.37 \times 10^5\ \mathrm{cm}^{-1}$ presents the lower limit. Further increasing the frequency, no propagating PhP solutions are found until at 7.132 THz (237.9 cm^{-1}) a new mode occurs in area B that is perpendicular to the mode in area A. Here, the momentum at the boundary $k_x = 1.66 \times 10^5\ \mathrm{cm}^{-1}$ is smaller than the limit of k_y in area A.

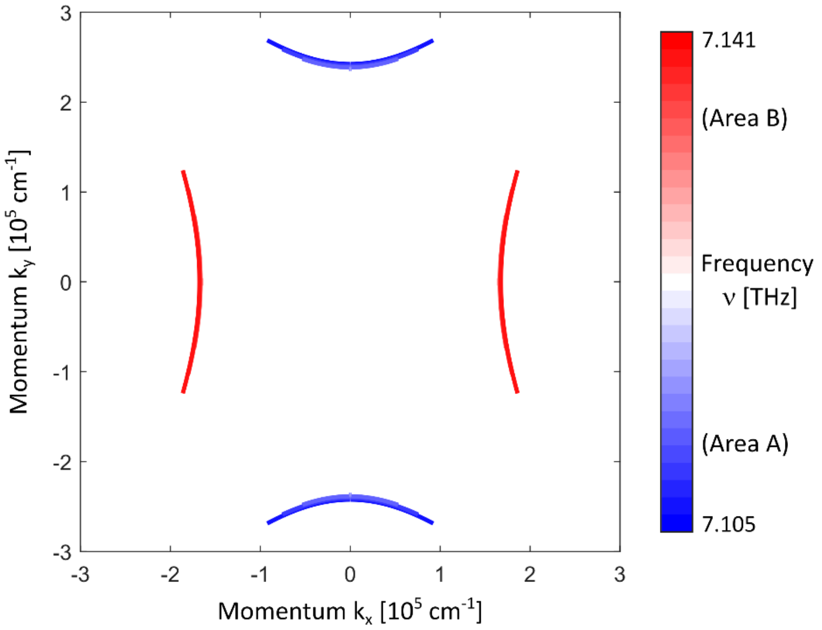

Figure S10. Rotation of the propagating l = 1 PhP mode in vicinity of the strong z-phonon TO frequency $\nu_{TO,[001],1} \approx 7.105$ THz (237 cm^{-1}). Frequency dependence of the (analytical) IFCs with l = 1 and $Re(k) > Im(k)$ around the zero-crossing of $Re(\varepsilon_z)$. Note that the position of sign change is slightly shifted towards higher frequencies from the nominal $\nu_{TO,[001],1}$. Propagating solutions within area A are found up until 7.114 THz with the hyperbolas major axis aligned along y-direction (= [100] direction) with the minimum in-plane momentum of $k_y = 2.37 \times 10^5 \mathrm{cm}^{-1}$. Increasing the frequency, the propagating modes re-emerge at 7.132 THz, now with the hyperbola's main axis aligned along x-direction (= [100] direction) with the momentum of $k_x = 1.66 \times 10^5 \mathrm{cm}^{-1}$.

In conclusion, biaxial GeS in the studied frequency range proves to be an excellent material to study the influence of the interplay between the in-plane and out-of-plane permittivity on the different PhP modes. In particular the case of the two hyperbolic l = 0, 1 modes aligned

perpendicular to each other (that was earlier predicted theoretically for such an exotic permittivity[13]) is of fundamental interest, while experimental proof is still missing.

Note S9: Natural canalization of PhPs in single slabs of GeS at THz frequencies

In this section we demonstrate that it is possible to achieve canalization of PhPs in single slabs of GeS placed on top of HRFZ Si substrates at THz frequencies. To do so, we first calculate the spatial distribution of the vertical component of the electric near field, $Re[E_z(x,y)]$, launched in the heterostructures by a vertical electric point dipole at a frequency ν_C = 8.57 THz (**Figure S11**a, reproduced from Figure 3d). PhPs propagate within a hyperbolic sector centered along the [100] crystal direction. Due to the extreme narrowing of the hyperbolic sector, PhPs propagate in their majority along one specific direction (canalization regime). On the other hand, elliptical wavefronts appear faintly in the neighborhood of the origin.

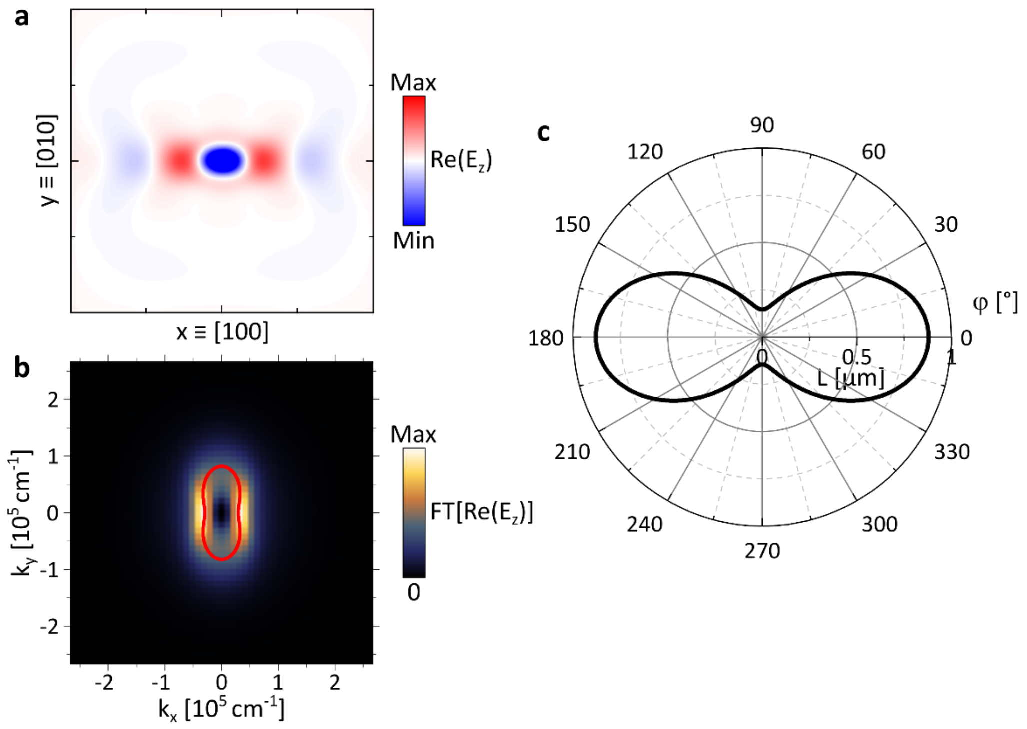

Figure S11. Natural canalization of PhPs in single slabs of GeS. a) Simulated real-space field distribution $Re[E_z(x,y)]$ at the GeS/air interface at ν_C = 8.57 THz. b) Fourier transform of the real space image in (a) (false color plor) and analytical polaritonic IFC (red line) reproduced from Figure 3d. c) Analytical polaritonic propagation length L as a function of the in-plane angle φ.

To study the canalization regime in detail, we analyze the polaritonic IFC in momentum space. To that end, we perform the Fourier transform (FT) of the near-field $Re[E_z(x,y)]$ simulation shown in Figure S11a (reproduced from Figure 3d). The FT is a closed curve, revealing both a hyperbolic-like shape IFC along the [100] direction and an elliptical-like shape along the [010] direction. Note that the IFC exhibits a strong flattening along the [100] direction, explaining the canalization of PhPs along this direction. Moreover, the numerical IFC (color plot) in Figure S11b shows an excellent agreement with the analytical curve given by Equation (1) in the main text (red line).

To further characterize the canalization of PhPs in GeS, we study the polaritonic damping along all in-plane directions. Figure S11c shows the polaritonic propagation length L, calculated from $L = Im(k)^{-1}$, where k is obtained as a function of φ from Equation (1). φ is the angle that k forms with the x-axis (or, equivalently, the [100] crystal axis). We observe the maximum value for L along the canalization direction, unambiguously demonstrating canalization of propagating PhPs along this direction.

From a practical perspective, our results demonstrate canalization of PhPs in natural single crystal slabs. Notice that up to now the canalization has only been demonstrated in twisted stacks of crystal slabs.[14–17]

Note S10: Impact of the FEL spectral bandwidth on PhP properties

When employing a pulsed FEL with finite spectral bandwidth and picosecond pulse length in polariton interferometry, two effects of the exciting pulse on the measured polariton properties have to be considered:[18] On one hand, the FEL's ps pulse length may artificially shorten the measured PhP life time (in case $\tau_{FEL} < \tau_{PhP}$). On the other hand, the FEL pulse with considerable spectral bandwidth $\Delta\nu$ excites (according to the shape of the dispersion) a bunch of polaritons

with varying momentum and group velocity. In the following, both independent effects are addressed in detail.

FEL pulse length: In this work, we perform polariton interferometry applying a selfhomodyne detection scheme in combination with a pulsed light source. Due to the FEL's 13 MHz repetition rate, each pulse can be treated individually, as two successive pulses are about 77 ns apart (equal to a distance of 23 m in air). In the experiment, the front of the FEL pulse (in time) excites the s-SNOM tip, which in turn launches PhPs that propagate across the GeS flake with momentum k and group velocity $v_g << c$. A portion of the PhPs gets reflected at an edge and travels back to the tip, where it coincides with the field of the tip polarized by the tail of the same FEL pulse. The polariton's electric field is then picked up by the tip and scattered into the far-field for the purpose of detection. The light intensity measured at the detector, however, is constituted by near- and far-field contributions, with the dominant term at a demodulation order of $n = 2$ being $I(2\Omega) \cong |E_{bg}| \times |E_{NF,2\Omega}| \cos(\varphi_{NF,2\Omega} - \varphi_{bg})$ ('multiplicative background'), as $|E_{NF}|^2$ is negligible.[19] The background contribution is directly scattered off the tip (without any near-field interaction) and, hence, shows the same time signature as the FEL pulse. This leads to the inherent condition, that the experimentally observable PhP life time cannot exceed the FEL pulse length, as the E_{bg} contribution is essential for the polariton field detection. Note that we anticipate the front of the FEL pulse to excite the (detected) polaritons that later interfere with the tail of the FEL pulse at the position of the tip. In case of GeS, we find the polariton life times $\tau_{PhP} < 2.5$ ps falling well below the lower limit of the FEL pulse length of about $\tau_{FEL} \gtrsim 5$ ps. Hence, the finite FEL pulse length does not present a limitation to the PhP life times measured experimentally. Moreover, note that τ_{FEL} represents the full width at half maximum of an (idealized) Gaussian pulse and thus, (weaker) field oscillations are still present at the tip, even after the nominal pulse length.

FEL spectral bandwidth: Secondly, the FEL pulse's spectral bandwidth can have a considerable impact on the perceived momentum (mostly *Im*[*k*]) depending on the local shape of the dispersion curve. The FEL pulse in frequency space ideally holds a Gaussian shape with a particular bandwidth $\Delta\nu_{FEL}$ and a center frequency ν_{FEL}. When inserting this distribution (ν_{FEL}, $\Delta\nu_{FEL}$) of exciting frequencies into the GeS dispersion, we find polaritons with a range of (complex) momentum k and group velocity v_g to be launched simultaneously by a single FEL pulse. In the polariton interferometry experiment, at the detector we measure the superposition of this distribution of PhPs, which is characterized by destructive interference effects, especially at larger tip-edge distances, where the phase of different PhPs could become even inverted. Thus, the resulting effective PhP *Im*(*k*) generally decreases with increasing FEL bandwidth.

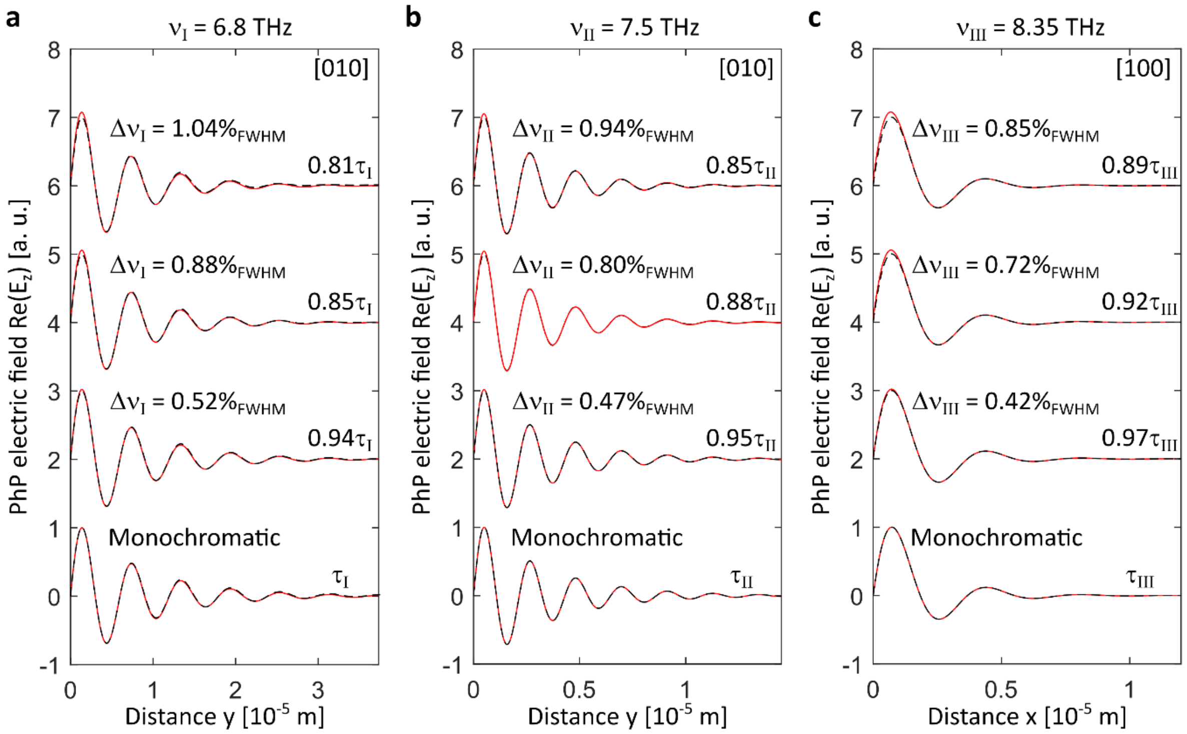

Figure S12. Modelled 2D PhP field distribution for a 224 nm-thick GeS slab for varying excitation bandwidths $\Delta\nu_{FEL}$ = 0, 0.035, 0.06, and 0.07 THz. For FEL pulses with center frequency ν_i and bandwidth $\Delta\nu_i$ the excited effective PhP electric fields are calculated applying the analytical model (Equation (1); black dashed lines, curves offset for visibility). The E_z-field distributions are fitted and the corresponding life times extracted (red curves). For FEL bandwidths representative for our experiments (typically $\Delta\nu$ = 0.6 – 0.9%$_{FWHM}$), we find life times artificially reduced by roughly 10 – 15%.

To estimate the magnitude of the described effect for the PhPs in GeS, we insert the $\Delta\nu_{FEL}$ into the analytical model and fit the resulting superposed electric field to obtain the effective PhP momentum (**Figure S12**). Here, for the center frequencies of ν_{FEL} = 6.8, 7.5, and 8.35 THz (similar to Note S6 and S7) we calculate the polariton life time for FEL spectral bandwidths of $\Delta\nu_{FEL}$ = 0, 0.035, 0.06, and 0.07 THz. The black dashed curves represent the calculated effective polariton field E_z and the red curves correspond to the fittings used to obtain the momentum and life time. At the center frequencies investigated and for relative FEL spectral bandwidths of $\Delta\nu_{FEL}$ = 0.6 - 0.9 $\%_{FWHM}$ (typical values for the FEL in our experiment) we find a reduction of the experimental life times of roughly 10 – 15 % as compared to monochromatic excitation. Note that these values present an estimate of a systematic error that is otherwise disregarded when fitting the experimental $S_{2\Omega}$ profiles.

In summary, as a consequence of the FEL spectral bandwidth, the actual PhP life times in GeS may be slightly longer than deduced from our experiment. Further note that the impact of FEL bandwidth is expected to be more significant in spectral areas where the dispersion is (i) highly nonlinear or (ii) has a large slope dν / dk, respectively.

Note S11: PhP group velocity v_g

The PhP group velocity v_g describes the travelling speed of the envelope of the tip- or edge-launched PhP wave packet and is typically highly frequency dependent. Moreover, in anisotropic materials it may hold different values for PhP propagation along different directions. Generally, the group velocity is calculated from the PhP dispersion ν(k) using the relation $v_g = 2\pi c\ d\omega/dk$. For phonon or plasmon polaritons, the group velocity is normally significantly slower than the speed of light, which is due to the strong coupling between the light and the excitation in matter (*i.e.*, the light field dragging the surface charges or lattice displacements). For the 224 nm-thick GeS flake, the calculated PhP group velocities are

demonstrated in **Figure S13**. Notably, the anisotropic group velocity has poles where the derivative of the dispersion ν(k) changes sign and is negative within the areas of anomalous dispersion.

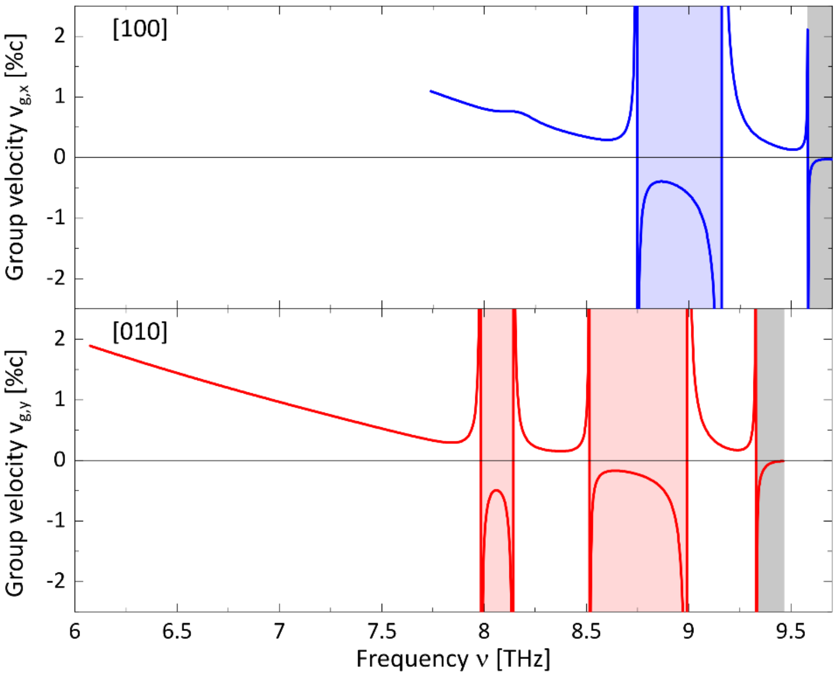

Figure S13. PhP group velocities along [100] (top panel) and [010] direction (bottom panel) calculated from the analytical dispersion curve. The red- and blue-shaded areas mark the areas where the dispersion is anomalous in the different back bending regimes. The grey-shade denote the group velocity becoming negative near the high-frequency limit of the in-plane reststrahlen bands (*i.e.*, the LO modes), which is typical for phonon polaritons.

REFERENCES

(1) Tan, D.; Lim, H. E.; Wang, F.; Mohamed, N. B.; Mouri, S.; Zhang, W.; Miyauchi, Y.; Ohfuchi, M.; Matsuda, K. Anisotropic Optical and Electronic Properties of Two-Dimensional Layered Germanium Sulfide. *Nano Res.* **2017**, *10*, 546–555.

(2) Chandrasekhar, H. R.; Humphreys, R. G.; Cardona, M. Pressure Dependence of the Raman Spectra of the IV-VI Layer Compounds GeS and GeSe. *Phys. Rev. B* **1977**, *16*, 2981–2983.

(3) Gashimzade, F. M.; Guseinova, D. A.; Jahangirli, Z. A.; Nizametdinova, M. A. Ab Initio Calculation of Vibrational Spectra of Orthorhombic IV–VI Layered Crystals. *Phys. Solid State* **2013**, *55*, 1802–1807.

(4) Wiley, J. D.; Buckel, W. J.; Schmidt, R. L. Infrared Reflectivity and Raman Scattering in GeS. *Phys. Rev. B* **1976**, *13*, 2489.

(5) Sutter, E.; Zhang, B.; Sun, M.; Sutter, P. Few-Layer to Multilayer Germanium(II) Sulfide: Synthesis, Structure, Stability, and Optoelectronics. *ACS Nano* **2019**, *13*, 9352–9362.

(6) Yu, L.-M.; Degiovanni, A.; Thiry, P. A.; Ghijsen, J.; Caudano, R.; Lambin, P. Infrared Optical Constants of Orthorhombic IV-VI Lamellar Semiconductors Refined by a Combined Study Using Optical and Electronic Spectroscopies. *Phys. Rev. B* **1993**, *47*, 16222.

(7) Li, P.; Dolado, I.; Alfaro-Mozaz, F. J.; Nikitin, A. Y.; Casanova, F.; Hueso, L. E.; Vélez, S.; Hillenbrand, R. Optical Nanoimaging of Hyperbolic Surface Polaritons at the Edges of van Der Waals Materials. *Nano Lett.* **2017**, *17*, 228–235.

(8) Woessner, A.; Lundeberg, M. B.; Gao, Y.; Principi, A.; Alonso-González, P.; Carrega, M.; Watanabe, K.; Taniguchi, T.; Vignale, G.; Polini, M.; Hone, J.; Hillenbrand, R.; Koppens, F. H. L. Highly Confined Low-Loss Plasmons in Graphene–Boron Nitride Heterostructures. *Nat. Mater.* **2015**, *14*, 421–425.

(9) Giles, A. J.; Dai, S.; Vurgaftman, I.; Hoffman, T.; Liu, S.; Lindsay, L.; Ellis, C. T.; Assefa, N.; Chatzakis, I.; Reinecke, T. L.; Tischler, J. G.; Fogler, M. M.; Edgar, J. H.; Basov, D. N.; Caldwell, J. D. Ultralow-Loss Polaritons in Isotopically Pure Boron Nitride. *Nat. Mater.* **2018**, *17*, 134–139.

(10) Bylinkin, A.; Schnell, M.; Autore, M.; Calavalle, F.; Li, P.; Taboada-Gutièrrez, J.; Liu, S.; Edgar, J. H.; Casanova, F.; Hueso, L. E.; Alonso-Gonzalez, P.; Nikitin, A. Y.; Hillenbrand, R. Real-Space Observation of Vibrational Strong Coupling between Propagating Phonon Polaritons and Organic Molecules. *Nat. Photonics* **2021**, *15*, 197–202.

(11) Dai, S.; Fei, Z.; Ma, Q.; Rodin, A. S.; Wagner, M.; McLeod, A. S.; Liu, M. K.; Gannett, W.; Regan, W.; Watanabe, K.; Taniguchi, T.; Thiemens, M.; Dominguez, G.; Neto, A. H. C.; Zettl, A.; Keilmann, F.; Jarillo-Herrero, P.; Fogler, M. M.; Basov, D. N. Tunable Phonon Polaritons in Atomically Thin van Der Waals Crystals of Boron Nitride. *Science* **2014**, *343*, 1125–1129.

(12) Duan, J.; Álvarez-Pérez, G.; Voronin, K. V.; Prieto, I.; Taboada-Gutiérrez, J.; Volkov, V. S.; Martín-Sánchez, J.; Nikitin, A. Y.; Alonso-González, P. Enabling Propagation of Anisotropic Polaritons along Forbidden Directions via a Topological Transition. *Sci.*

Adv. **2021**, *7*, eabf2690.

(13) Álvarez-Pérez, G.; Voronin, K. V.; Volkov, V. S.; Alonso-González, P.; Nikitin, A. Y. Analytical Approximations for the Dispersion of Electromagnetic Modes in Slabs of Biaxial Crystals. *Phys. Rev. B* **2019**, *100*, 235408.

(14) Chen, M.; Lin, X.; Dinh, T. H.; Zheng, Z.; Shen, J.; Ma, Q.; Chen, H.; Jarillo-Herrero, P.; Dai, S. Configurable Phonon Polaritons in Twisted α-MoO_3. *Nat. Mater.* **2020**, *19*, 1307–1311.

(15) Duan, J.; Capote-Robayna, N.; Taboada-Gutiérrez, J.; Álvarez-Pérez, G.; Prieto, I.; Martín-Sánchez, J.; Nikitin, A. Y.; Alonso-González, P. Twisted Nano-Optics: Manipulating Light at the Nanoscale with Twisted Phonon Polaritonic Slabs. *Nano Lett.* **2020**, *20*, 5323–5329.

(16) Hu, G.; Ou, Q.; Si, G.; Wu, Y.; Wu, J.; Dai, Z.; Krasnok, A.; Mazor, Y.; Zhang, Q.; Bao, Q.; Qiu, C.-W.; Alù, A. Topological Polaritons and Photonic Magic Angles in Twisted α-MoO_3 Bilayers. *Nature* **2020**, *582*, 209–213.

(17) Zheng, Z.; Sun, F.; Huang, W.; Jiang, J.; Zhan, R.; Ke, Y.; Chen, H.; Deng, S. Phonon Polaritons in Twisted Double-Layers of Hyperbolic van Der Waals Crystals. *Nano Lett.* **2020**, *20*, 5301–5308.

(18) de Oliveira, T. V. A. G.; Nörenberg, T.; Álvarez-Pérez, G.; Wehmeier, L.; Taboada-Gutiérrez, J.; Obst, M.; Hempel, F.; Lee, E. J. H.; Klopf, J. M.; Errea, I.; Nikitin, A. Y.; Kehr, S. C.; Alonso-González, P.; Eng, L. M. Nanoscale-Confined Terahertz Polaritons in a van Der Waals Crystal. *Adv. Mater.* **2021**, *33*, 2005777.

(19) Dai, G.; Yang, Z.; Geng, G.; Li, M.; Chang, T.; Wei, D.; Du, C.; Cui, H.-L.; Wang, H. Signal Detection Techniques for Scattering-Type Scanning near-Field Optical Microscopy. *Appl. Spectrosc. Rev.* **2018**, *53*, 806–835.
